# Predictive potentials of glycosylation-related genes in glioma prognosis and their correlation with immune infiltration

**DOI:** 10.1038/s41598-024-51973-0

**Published:** 2024-02-23

**Authors:** Yi-Fei Sun, Lan-Chun Zhang, Rui-Ze Niu, Li Chen, Qing-Jie Xia, Liu-Lin Xiong, Ting-Hua Wang

**Affiliations:** 1 Department of Neurosurgery, West China Hospital, Sichuan University, Chengdu, 610041 Sichuan China; 2 Laboratory Animal Department, Kunming Medical University, Kunming, 650031 Yunnan China; 3 Institute of Neurological Disease, West China Hospital, Sichuan University, No. 17, Section 3 of South Renmin Road, Chengdu, 610041 China; 4 Translational Neuromedicine Laboratory, Affiliated Hospital of Zunyi Medical University, No. 149, Dalian Road, Zunyi, 563000 Guizhou China

**Keywords:** Biological techniques, Cancer

## Abstract

Glycosylation is currently considered to be an important hallmark of cancer. However, the characterization of glycosylation-related gene sets has not been comprehensively analyzed in glioma, and the relationship between glycosylation-related genes and glioma prognosis has not been elucidated. Here, we firstly found that the glycosylation-related differentially expressed genes in glioma patients were engaged in biological functions related to glioma progression revealed by enrichment analysis. Then seven glycosylation genes (*BGN*, *C1GALT1C1L*, *GALNT13*, *SDC1*, *SERPINA1*, *SPTBN5* and *TUBA1C*) associated with glioma prognosis were screened out by consensus clustering, principal component analysis, Lasso regression, and univariate and multivariate Cox regression analysis using the TCGA-GTEx database. A glycosylation-related prognostic signature was developed and validated using CGGA database data with significantly accurate prediction on glioma prognosis, which showed better capacity to predict the prognosis of glioma patients than clinicopathological factors do. GSEA enrichment analysis based on the risk score further revealed that patients in the high-risk group were involved in immune-related pathways such as cytokine signaling, inflammatory responses, and immune regulation, as well as glycan synthesis and metabolic function. Immuno-correlation analysis revealed that a variety of immune cell infiltrations, such as Macrophage, activated dendritic cell, Regulatory T cell (Treg), and Natural killer cell, were increased in the high-risk group. Moreover, functional experiments were performed to evaluate the roles of risk genes in the cell viability and cell number of glioma U87 and U251 cells, which demonstrated that silencing *BGN*, *SDC1*, *SERPINA1*, *TUBA1C*, *C1GALT1C1L* and *SPTBN5* could inhibit the growth and viability of glioma cells. These findings strengthened the prognostic potentials of our predictive signature in glioma. In conclusion, this prognostic model composed of 7 glycosylation-related genes distinguishes well the high-risk glioma patients, which might potentially serve as caner biomarkers for disease diagnosis and treatment.

## Introduction

Glioma is the most common primary CNS malignancies that can be divided into two types based on the malignancy of the tumor cells: low-grade gliomas (WHO grade 1 to 2) and high-grade gliomas (WHO grade 3 to 4), with a high mortality rate despite having a low incidence compared to other malignancies^1,2^. Glioblastoma (WHO grade 4) is the most common and malignant histologic type of glioma, accounting for 49.1% of all CNS malignancies. It is more common in men than women, with incidence increasing with age, and its extremely aggressive nature causes rapid tumor spread to other brain regions, resulting in diffuse infiltrative growths^3,4^. Histological and immunohistochemical characteristics have traditionally been used to grade central system tumors. Glioma biology studies in recent years have discovered a variety of critical genetic and molecular foundations that can influence patient prognosis^5^, such as IDH mutational status, 1p/19q co-deletion, histone H3-K27M alterations, and MGMT promoter methylation^6^. One of the most common types of post-translational modifications is glycosylation. Glycosylation modification is the process of attaching monosaccharides or glycans to certain amino acid residues in glycoproteins under the supervision of an enzyme reaction^7^. N-linked glycosylation, O-linked glycosylation, C-glycosylation, and glycosyl-phosphatidyl inositol (GPI)-mediated glycosylation are the four primary types of protein glycosylation modifications^8^. The two most common enzymatic protein glycosylation processes that occur in the endoplasmic reticulum and Golgi apparatus, as well as the glycosylated forms of most cell surface receptors and secreted proteins, such as extracellular matrix glycoproteins, cytokines, growth factors, and cell surface proteins, are N-linked glycosylation and O-linked glycosylation, respectively^9^. Tumor cells overexpress numerous glycans on the cell surface by mucin secretion, and specific glycans can impact the spatial folding and stability of proteins, accelerating tumorigenesis and progression. Modified glycans function as receptors or adhesion molecules, aiding various facets of tumor growth such as tumor cell proliferation, differentiation, migration, and tumor angiogenesis and invasion^10^. The glycosylation process is regulated by the interaction of glycosyltransferases and glycosidases, and there are limited investigations on the role of regulatory variables related with glycosylation alterations in glioma prognosis. Some glycosylation genes have been linked to malignant changes in different differentiation states and are significantly more expressed in undifferentiated brain tumor cells than in differentiated cells, suggesting that these genes may play a role in inhibiting brain tumor stem cell differentiation and could be used as biomarkers to assess glioma aggressiveness^11^. In another study, an N-linked glycosylation small molecule inhibitor (*NGI-1*) was able to reduce glycosylation and activation of receptor tyrosine kinases to inhibit tumor cell growth and increase epidermal growth factor receptor family activation, improving glioma cell sensitivity to radiotherapy and chemotherapeutic agent cytotoxicity^12^. Together, these research demonstrate that dysregulation of glycosylation alterations can promote cancer development and that some glycosylation-associated RNA modifications can have a negative impact on glioma patients' prognosis.

In this study, we used genomic bioinformatics to assess the expression levels of glycosylation modification-related genes in gliomas and validate the value of our established glycosylation-related features for glioma prognosis, provide novel insights into development of new non-invasive biomarkers.

## Materials and methods

### Glycosylation-related gene acquisition

The Molecular Signature Database (https://www.gsea-msigdb.org/gsea/msigdb) was used to download the set of genes related to glycosylation modifications: The gene members of GOBP_GLYCOSYLATION (M15710), GOBP_PROTEIN_N_LINKED_GLYCOSYLATION (M10780), REACTOME_ASPARAGINE_N_LINKED_GLYCOSYLATION (M894), REACTOME_DISEASES_OF_GLYCOSYLATION (M27275), and REACTOME_O_LINKED_ GLYCOSYLATION_OF_MUCINS (M546). Finally, a total of 552 glycosylation-related genes were selected after removing duplicates.

### Acquisition and collation of gene expression matrix and clinicopathological data

First, gene expression sequencing of 689 glioma samples from TCGA (LGG: n = 523, GBM: n = 166) and 5 paraneoplastic samples from TCGA and 1152 normal brain tissues from GTEx database were downloaded from University of California Santa Cruz (UCSC) Data. To produce gene expression matrices for 1846 samples, the data was log2 (TPM + 1) processed and gene ID transformed. We extracted clinical case factors of glioma patients from the TCGA and cBioPortal databases (https://www.cbioportal.org/), respectively, and extracted survival outcome, survival time, age, gender, tumor grade, histological subtype, 1p/19q co-deletion status, radiotherapy status, MGMT promoter methylation, and IDH mutation status. The data information of glioma patients in the Chinese Glioma Genome Atlas (CGGA) database was used as external validation, and the data information of 1018 glioma cases with dataset IDs mRNAseq_693 and mRNAseq_325 and dataset ID mRNA sequencing (non- glioma as control) of 20 non-tumor brain tissues to obtain the gene expression profile matrix. To eliminate batch effects, the data was processed using the "limma" and "sva" packages.

### Differential analysis and clustering analysis of glycosylation-related regulators

Following that, we looked at the differential expression of glycosylation-related regulatory genes in glioma and normal brain tissues, using the following criteria: adj-P < 0.05 and differential multiplicity |Log2FC > 1| were regarded differently expressed. We used violin plots and expression heat maps to plot correlation analyses for genes with differential multiplicity > 2. Consensus clustering is a data mining technique for identifying a group of unknown genomes that share biological characteristics. We split 689 glioma patients into two different subgroups and used PCA master analysis using the "limma," "ConsensusClusterPlus," and "scatterplot3d" packages to confirm the reliability of the consensus clustering results.

### Enrichment analysis and survival analysis

The functional annotation of differentially expressed genes between the two groupings was done using Gene Ontology (GO) and the Kyoto Encyclopedia of Genes and Genomes (KEGG)^13^, and Kaplan–Meier survival analysis was performed to assess differences in survival rates and plot survival curves. The packages "clusterProfiler" and "survival" are used to carry out the analysis phases. The variations in clinicopathological factors between the two clusters were analyzed using the chi-square test, which was shown as a heat map.

### Prognostic model construction

The researchers conducted the univariate Cox regression analysis to look for glycosylation-related regulators linked to Overall Survival (OS) in glioma patients, with a p-value < 0.05 serving as the qualifying threshold. Model covariance was reduced using Last absolute shrinkage and selection operator (LASSO) regression andmultifactorial Cox regression analysis. Finally, a risk profile of glioma patients was created using seven glycosylation-related regulatory genes, and a sample risk score was calculated using the risk score method below, with high-low risk categorization based on each patient's median-risk score. The formula for calculating the risk score was as follows:

$$Risk\;score = \sum\nolimits_{i = 1}^{7} {\left( {Coef_{i} *Exp_{i} } \right)}$$

### Model evaluation and external validation

Glioma data from the CGGA database was used as an external validation cohort. Using the same risk score calculation procedure and risk cutoff values, patients were separated into high-risk and low-risk groups. The risk prediction model's prognostic value and external evaluation were assessed using Kaplan–Meier survival analysis and Receiver Operating Characteristic (ROC) curves analysis.

### Column line graph construction

We used the "survival" package to run univariate and multifactorial Cox regression analysis to examine independent risk variables and draw forest plots for glioma. To aid clinical analysis, risk scores were merged with other independent prognostic characteristics and prediction column line graphs of 1-, 3-, and 5-year mortality in glioma patients were created using the "rms" and "regplot" programs.

### Risk profile gene set enrichment analysis (GSEA)

Using GSEA (version 4.1.0) software, we performed enrichment analysis to learn more about the functions and pathways involved in glycosylation-related genes in glioblastoma. The normalized enrichment score^14^, nominal p-value (NOM p-val), and false discovery rate (FDR q-val) were our screening criteria, which were met when |NES|> 1, NOM p-val < 0.05, and FDR q-val < 0.25.

### Immunocorrelation analysis and clinical correlation analysis

To investigate the variations in immune responses between the two risk groups, we used the ssGSEA algorithm to evaluate the infiltration scores of 28 immune cells and immune-related pathways in the two groups based on the "GSVA" package's risk ratings. Because immune checkpoint expression levels may be related to immunotherapy response, the sensitivity of immune checkpoints was compared between high- and low-risk groups using the "ggpubr" package. A swarm map of associations between predicted genes, risk scores, and clinicopathological factors was created using the "beeswarm" package.

### Cell culturing

The U251 and U87 glioma tumor cell line, purchased from the Animal Zoology Department of Kunming Medical University, were cultured and used to generate derivatives. U251 and U87 cells were routinely grown in DMEM/high glucose (Hyclone, USA) containing 10% Foetal Bovine Serum (FBS; Hyclone, USA) and 1% penicillin–streptomycin solution (PSS, Hyclone, USA). After being washed with phosphate-buffered saline (PBS; Hyclone, USA) one or two times, the cells were digested for 2–3 min (min) with 0.25% trypsin (1–2 mL; Gibco). The cell digestion was terminated using complete medium. After centrifugation (800–1000 rpm for 5–8 min) and resuspension, the cell suspension was collected again and the cells were placed in a 25 t (3 mL) culture flask at a density of 4 × 10^5^ cells/mL and placed in the incubator.

### Small interfering RNA (siRNA) transfection assay

SiRNA is the most commonly used tool for gene silencing experiments, which can direct the RISC system to specifically shear the target RNA inside the cell thereby inhibiting the expression of the target gene. The siRNAs provided by RIBO are modified with 5'Chol + 2'OMe. Before transfection, riboFECT™ CP Buffer (1X) was prepared by diluting riboFECT™ CP Buffer (10X) using PBS. After removing the riboFECT™ CP Reagent, it was fully shaken in a vortex shaker and left at room temperature to return to room temperature before use. First, siRNA solutions were diluted in 1X riboFECT™ CP buffer to obtain final concentrations of 50, 75, and 100 nm. Next, RiboFECT ™ CP reagent was added to prepare the transfection complex, which was then added to the cell cultures. RNA was extracted 48 h after transfection for validation via RT-qPCR and the most efficient concentration was selected for formal experiments. The cell culture plates were incubated in a CO_2_ incubator at 37 °C for 48 h (h) for siRNA silencing effect assay and cell viability assay.

### Cell Counting Kit 8 (CCK8) assay

Cell viability after transfection was assayed by CCK-8. The cells were collected in the logarithmic growth phase, and the concentration of cell suspension was adjusted by adding 100 μL to each well of a 96-well plate to achieve a cell density of 3000 cells/well for the cells to be tested. The cells were incubated in 5% CO_2_, 37 °C incubator for 24 h. After transfecting the cells with different concentrations of interfering RNA, the plates were returned to the incubator and incubated for 48 h. After 10 μL CCK-8 solution was added to each well, incubation was continued for 2 h in the cell culture incubator, and the absorbance of each well was measured at 450 nm by an enzyme meter. Cell viability = [(experimental wells − blank wells)/(control wells − blank wells)] × 100%.

### Statistical analysis

All statistical analyses were performed using R software (version 4.2.1) and SPSS software (version 13.0; SPSS, Inc., Chicago, IL, USA). Data between the two groups were analysed using the Student’s *T* test. Data are presented as the mean ± standard deviation (SD). Statistical significance was set at *p* < 0.05.

## Results

### Identification of glycosylation-related regulators expressed in patients with glioma

**Figure 1:**
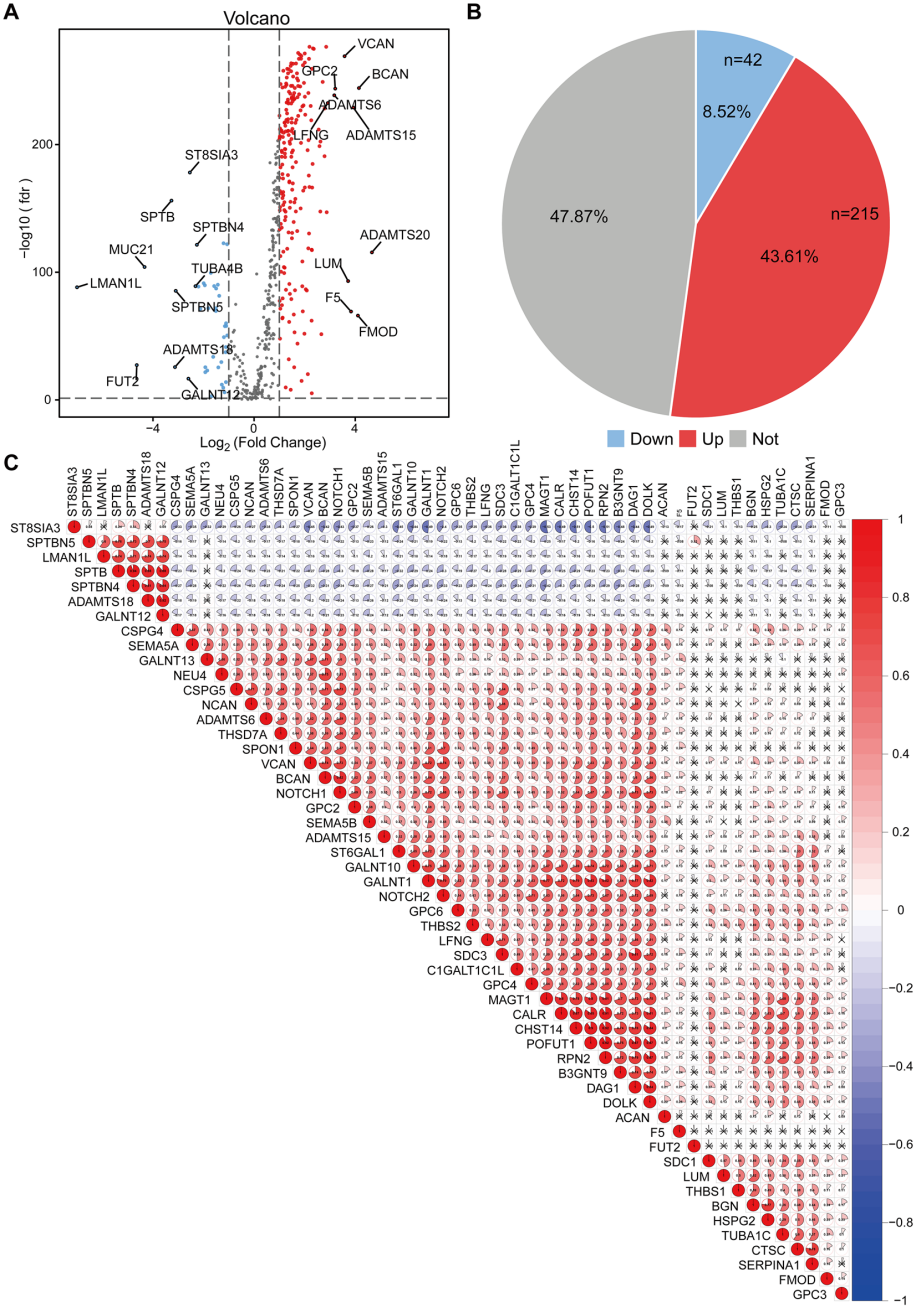
Differential analysis and correlation analysis of glycosylation genes. (**A**) Volcano plot of differential expression of glycosylation-related genes. Red: up-regulated genes; blue: down-regulated genes; gray: genes with no significant difference in expression. Conditions were met: (FDR) < 0.05 and |Log 2FC|> 1. (**B**) Pie charts of the proportion of up- and down-regulated genes. Red: up-regulated genes; blue: down-regulated genes. (**C**) Heat map of correlation between significantly different genes. Red: positive correlation; blue: negative correlation. Numbers in the circles indicate the correlation coefficients between genes.

**Figure 2:**
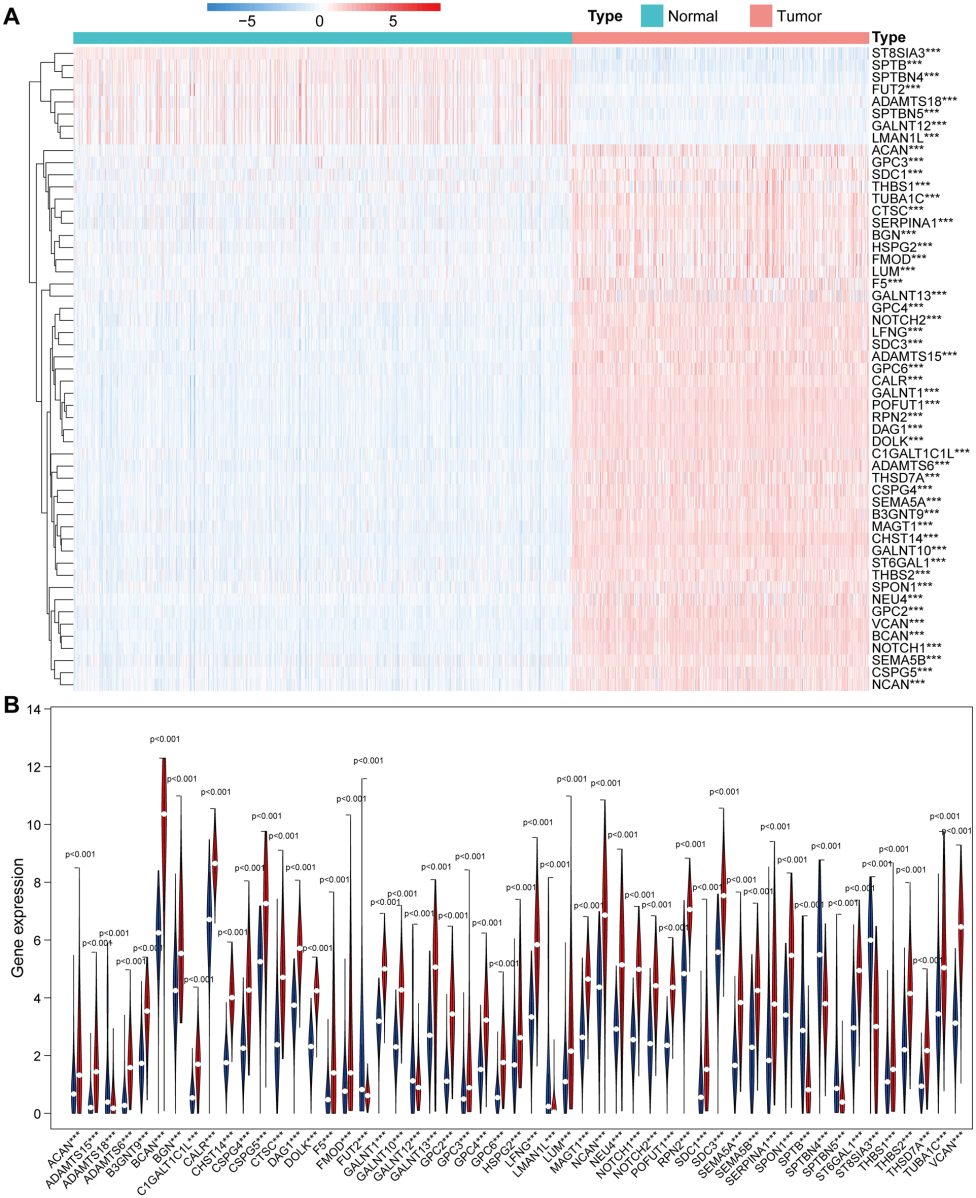
Differential expression of glycosylation genes in gliomas. (**A**) Heat map of differential expression of glycosylation-related genes. N: normal tissue; T: glioma tissue; red: high expression; blue: low expression. (**B**) Differential expression of glycosylation genes in glioma tissues and control tissues violin plot. Red: tumor tissues; blue: normal tissues.

First, 552 glycosylation-related genes were chosen for follow-up studies by downloading glycosylation-related regulators in five gene sets from the MSigDB database. After deleting duplicate genes, a total of 552 glycosylation-related genes were selected for follow-up investigations (Supplementary Table 1). We used the TCGA-GTEx database to compare the expression of 552 glycosylation-related genes in gliomas (n = 689) and non-tumor brain tissues (n = 1157), finding that 43.61% (n = 215) were up-regulated genes and 8.52% (n = 42) were down-regulated genes, with the top ten up- and down-regulated genes marked in the volcano plot (Fig. 1A,B). Following that, we chose 53 glycosylation-related genes in gliomas with |Log_2_FC > 2| for further investigation (Supplementary Table 2). The Pearson correlation analysis was used to look at the relationships between genes, and it found a strong positive correlation (0.94) between *SPTB* and *SPTBN4* and a strong negative correlation (− 0.53) between *ST8SIA3* and *MAGT1* (Fig. 1C). We can see that there are significant differences in the expression of glycosylation-related genes in both glioma and non-tumor brain tissues using the heat map and violin plot. Except for *LMAN1L*, *FUT2*, *SPTB*, *ADAMTS18*, *SPTBN5*, *GALNT12*, *ST8SIA3*, and *SPTBN4*, all glycosylation-related genes had considerably higher expression levels in tumors than in control tissues (*p* < 0.001, Fig. 2A,B).

### Consensus clustering of glycosylation-related regulators

**Figure 3:**
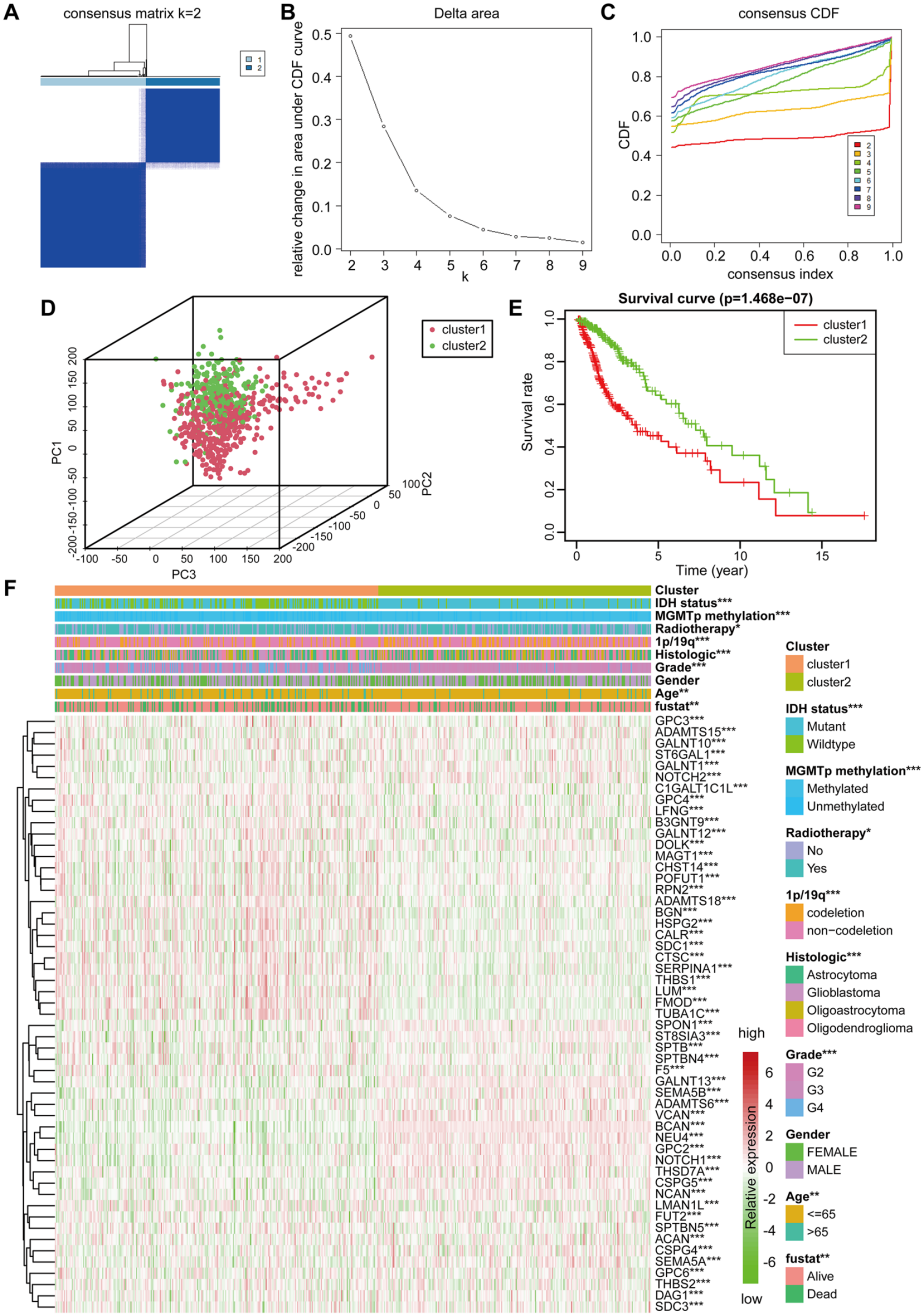
Clustering analysis and survival analysis of glioma patients based on glycosylated genes. (**A**) Consensus clustering for k = 2. (**B**) Plot of relative change in the area under the CDF curve for k = 1 ~ 9 falling stones. (**C**) CDF of k = 1 ~ 9 consensus clusters. (**D**) PCA analysis between cluster 1 and cluster 2. (**E**) Kaplan–Meier curves between cluster 1 and cluster 2 groups. (**F**) Heat map of multiple clinicopathological factors combined with glycosylated gene expression between cluster 1 and cluster 2, up-regulated in red and down-regulated in green. *p < 0.05, **p < 0.01 and ***p < 0.001. CDF: cumulative distribution function; PC: principal component.

We used the "ConsensusClusterPlus" package to perform a clustering analysis of the expression matrix of glycosylated genes in each sample to determine the ability of glycosylated genes to discriminate between glioma patients, and the results showed that the area under the Cumulative Distribution Function (CDF) curve with the area under the cluster count k = 2 was considered the best cluster (Fig. 3A–C). We separated the sample into two groups (cluster 1 and cluster 2) and used a PCA 3D pattern plot to show a clear separation between cluster 1 and cluster 2 (Fig. 3D). We ran a survival analysis of glioma patients in clusters 1 and 2 to see if glycosylation genes could differentiate between them. The survival analysis revealed that cluster2 glioma patients had a higher survival rate (*p* = 1.468e−07) than cluster 1 glioma patients (Fig. 3E), and the clinicopathological characteristics of the patients were also merged with the gene expression heat map (Fig. 3F), patients in clusters 1 and 2 had significant differences in WHO Grade, Histologic, 1p/19q status, MGMTp methylation, and IDH status (*p* < 0.001).

### GO and KEGG enrichment analysis

**Figure 4:**
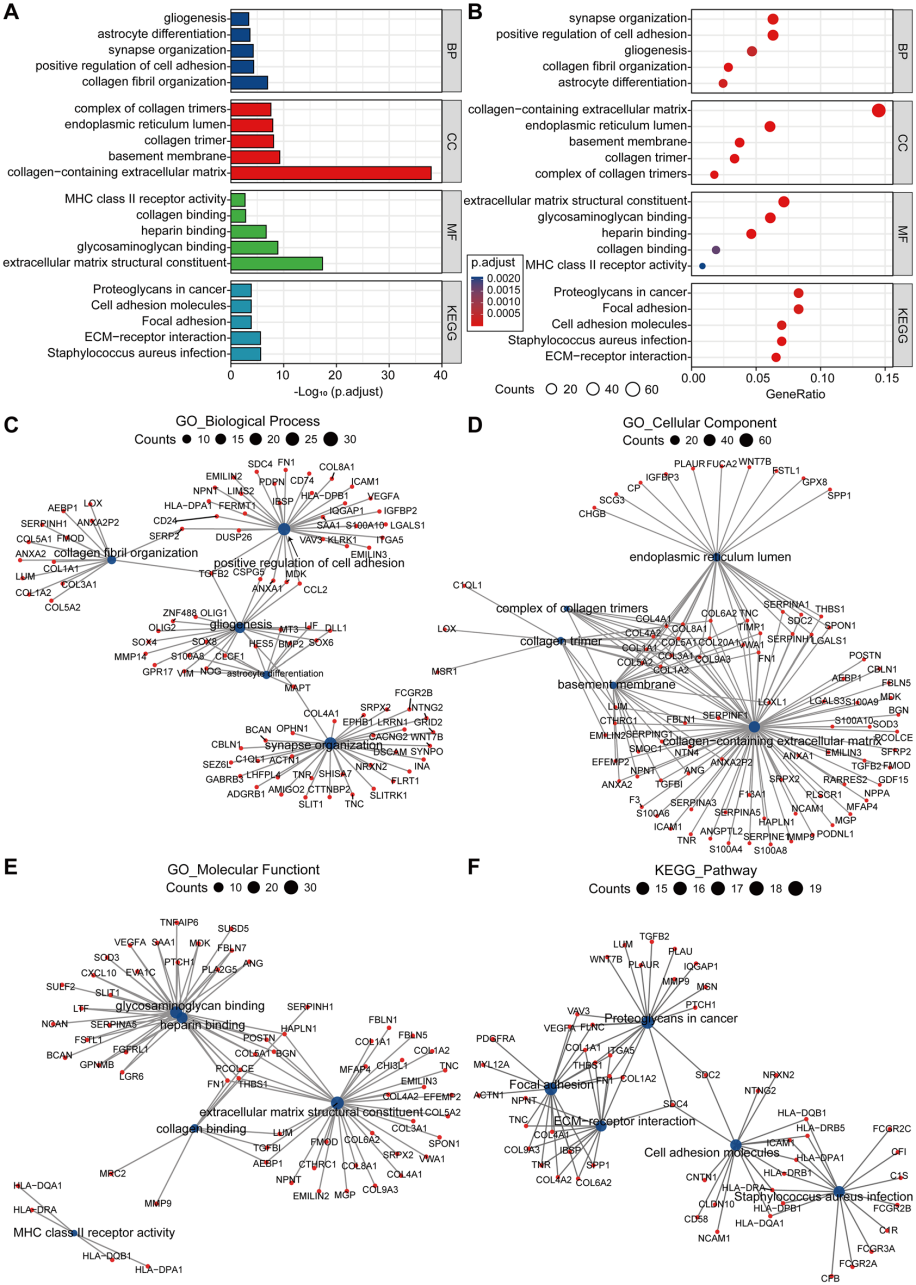
GO and KEGG enrichment analysis. (**A**) Bar graph of KEGG and GO enrichment analysis with − Log10 (p-adjust) as the horizontal coordinate. (**B**) Bubble plot of KEGG and GO enrichment analysis with the horizontal coordinate of the enrichment ratio GeneRatio. (**C**–**F**) Pathway-related gene network map of Biological Process, Cellular Component, Molecular Function and KEGG Pathway. BP: biological process; CC: cell component; MF: molecular function; GO: gene ontology; KEGG: Kyoto Encyclopedia of Genes and Genomes.

To investigate the biological differences between the two clusters, we did a difference analysis on the two clusters and screened out the difference genes that met the criteria of adj-*P* < 0.05 and |Log_2_FC > 1|, then analyzed the difference genes using GO and KEGG. The differential genes were mostly enriched in the "Proteoglycans in cancer," "ECM-receptor interaction," "Focal adhesion," "Cell adhesion molecules," and "Staphylococcus aureus infection" pathways, according to the KEGG analysis. The results of GO analysis showed that differential genes were significantly enriched in the biological processes such as "gliogenesis," "astrocyte differentiation," "collagen fibril organization," "synapse organization" and "positive regulation of cell adhesion". The differential genes were linked to cellular structures like "collagen-containing extracellular matrix," "collagen trimer," and "complex of collagen trimers," as well as molecular functions like "extracellular matrix structural constituent," "glycosaminoglycan binding," and "collagen binding" (Fig. 4A,B, Supplementary Table 3). In addition, we mapped the gene networks that are engaged in each pathway (Fig. 4C–F).

### Construction of a glycosylation-related gene prognostic risk model

**Figure 5:**
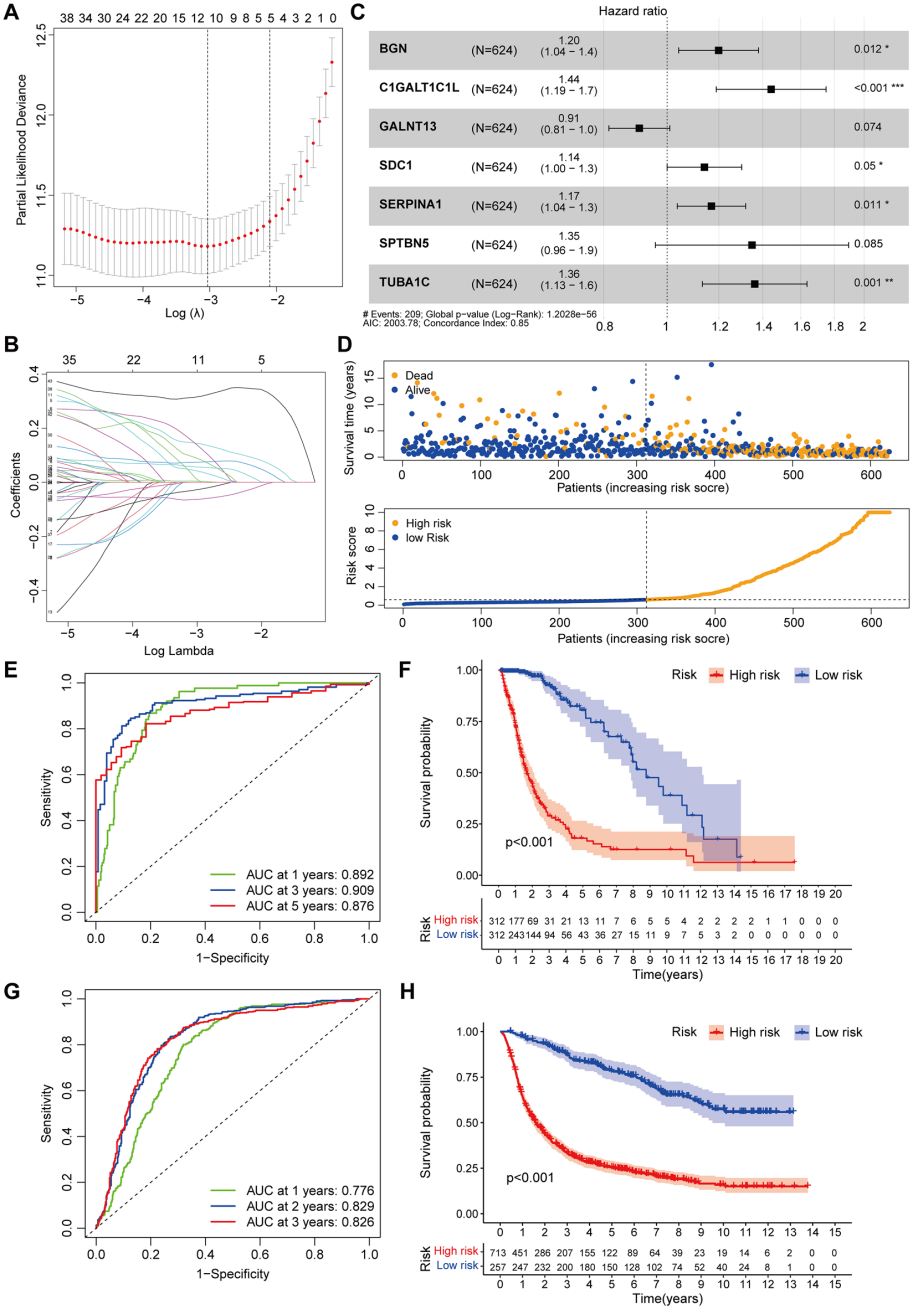
Prognostic modeling and evaluation of glioma risk. (**A**) Cross-validation for Lasso variable coefficient screening. (**B**) Trajectory plots of Lasso variables for prognosis-related glycosylation genes. (**C**) Multi-factor stepwise Cox regression analysis. (**D**) Scatter plot of survival status and risk score distribution of the model cohort patients. (**E**,**F**) Kaplan–Meier curves and 1-year, 3-year and 5-year ROC curves for OS of patients in the high-risk and low-risk groups of the TCGA-GTEx cohort. (**G**,**H**) Kaplan–Meier curves and 1-, 2-, and 3-year ROC curves for OS for patients in the high-risk and low-risk groups of the CGGA cohort. AUC: area under ROC curves.

We used the univariate Cox regression analysis to find 44 genes linked with OS in glioma patients (*p* < 0.05) to study the influence of glycosylation-related genes on prognosis (Supplementary Fig. 1). We attempted three machine learning methods to screen for differentially expressed genes for constructing prognostic models, and we were unable to select genes common to all three methods for prognostic modeling due to the small number of significantly different prognostic genes (Supplementary Table 4). Finally, Lasso regression analysis and stepwise Cox regression analysis were used to select seven genes linked with OS-related glycosylation for model development, including *BGN*, *C1GALT1C1L*, *GALNT13*, *SDC1*, *SERPINA1*, *SPTBN5*, and *TUBA1C*, all of which were risk factors (HR > 1) except GALNT13. The sample risk score was obtained based on the formula: risk score = regression coefficient * expression, i.e. (0.181 * BGN) + (0.366 * C1GALT1C1L)—(0.098 * GALNT13) + (0.131 * SDC1) + (0.156 * SERPINA1) + (0.299 * SPTBN5) + (0.308 * TUBA1C) (Fig. 5A–C).

### Evaluation and external validation of risk prognostic models

Patients were separated into two groups for prognostic analysis: low-risk (n = 312) and high-risk (n = 312) based on their median risk score. The survival time of glioma patients declined with rising risk scores, as evidenced in the scatter plot of survival status and the distribution of risk scores, and the survival time of high-risk patients was lower than that of low-risk patients (Fig. 5D). The area under the curve for 1-year, 3-year, and 5-year overall survival was 0.892, 0.909, and 0.876, respectively, indicating that the prediction model had strong predictive power for survival outcomes (Fig. 5E). The Kaplan–Meier curve of the risk-prognosis model revealed that patients in the high-risk group had a considerably lower survival rate (*p* < 0.001) than those in the low-risk group (*p* < 0.001, Fig. 5F). We used the CGGA database of glioma patients' gene expression profiles and survival information for external validation to see how accurate the prognostic model was. The same risk score algorithm was used to construct risk scores for each sample in the CGGA cohort, and patients were separated into high-risk (n = 713) and low-risk (n = 257) groups based on the risk threshold of the risk prediction model. The CGGA cohort had an area under the ROC curve of 0.776 at 1 year, 0.829 at 2 years, and 0.826 at 3 years (Fig. 5G). The Kaplan–Meier survival analysis revealed that high-risk patients had a considerably shorter overall survival (*p* < 0.001) than low-risk patients (Fig. 5H). The results of combining the CGGA and TCGA-GTEx cohorts revealed that the risk-prognosis model based on glycosylation-related regulators had good predictive potential for glioma patient survival. The expression heat map of the seven glycosylation-associated genes for which the model was built in combination with clinicopathological variables was plotted, and the high- and low-risk groups showed a substantial difference in gene expression (Supplementary Fig. 2).

### Model regression analysis and establishment of prognostic nomogram

**Figure 6:**
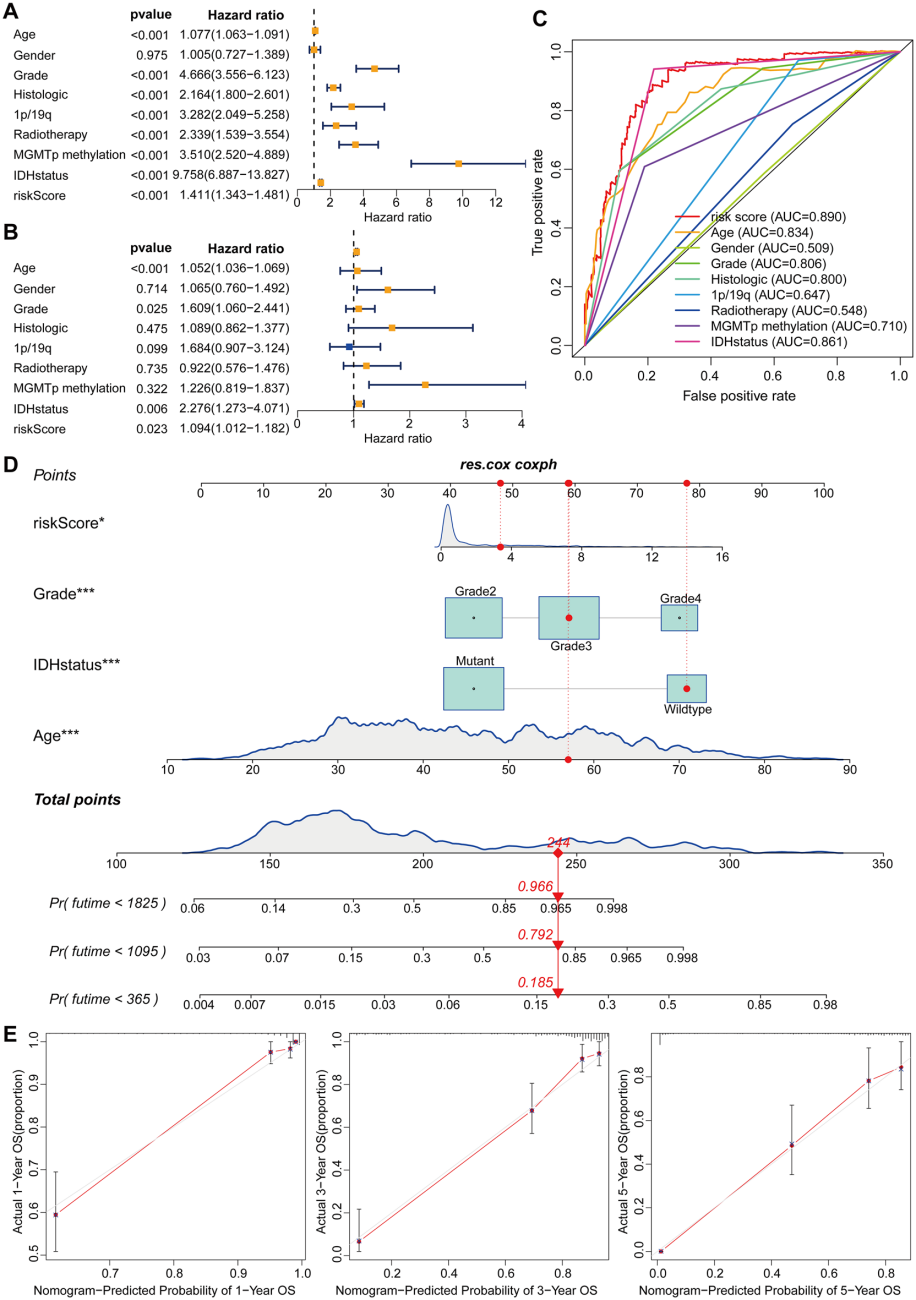
Single- and multi-factor Cox regression analysis and nomogram creation and calibration curves. (**A**) Forest plot of single-factor Cox regression analysis. (**B**) Forest plot of multi-factor Cox regression analysis. (**C**) Multivariate ROC curves. (**D**) The nomogram incorporating independent prognostic factors age, risk score, WHO Grade and IDH status to predict mortality in glioma patients. (**E**) The nomogram of 1-year, 3-year and 5-year calibration curves. *p < 0.05, ***p < 0.001. AUC: area under ROC curves.

We used univariate and multivariate Cox analysis to find that age, tumor grade, IDH status, and risk score are all independent prognostic variables for glioma patients (*p* < 0.05, Fig. 6A,B). The risk score was more accurate than other clinicopathological criteria for prediction, according to multivariate ROC curves (Fig. 6C). We constructed column line graphs of patient mortality at 1, 3, and 5 years to make it easier to forecast glioma patient survival. The nomogram included independent influencing factors such as age, tumor grade, IDH status, and risk score, and the concordance index (C-index) = 0.871 of the column line graphs revealed that risk score had a greater impact on prognosis in glioma patients than WHO Glioma Grade and IDH status (Fig. 6D). The 1-, 3-, and 5-year calibration curves in nomogram revealed good agreement between the nomogram risk model's predicted survival and actual survival (Fig. 6E).

### Gene set enrichment analysis

**Figure 7:**
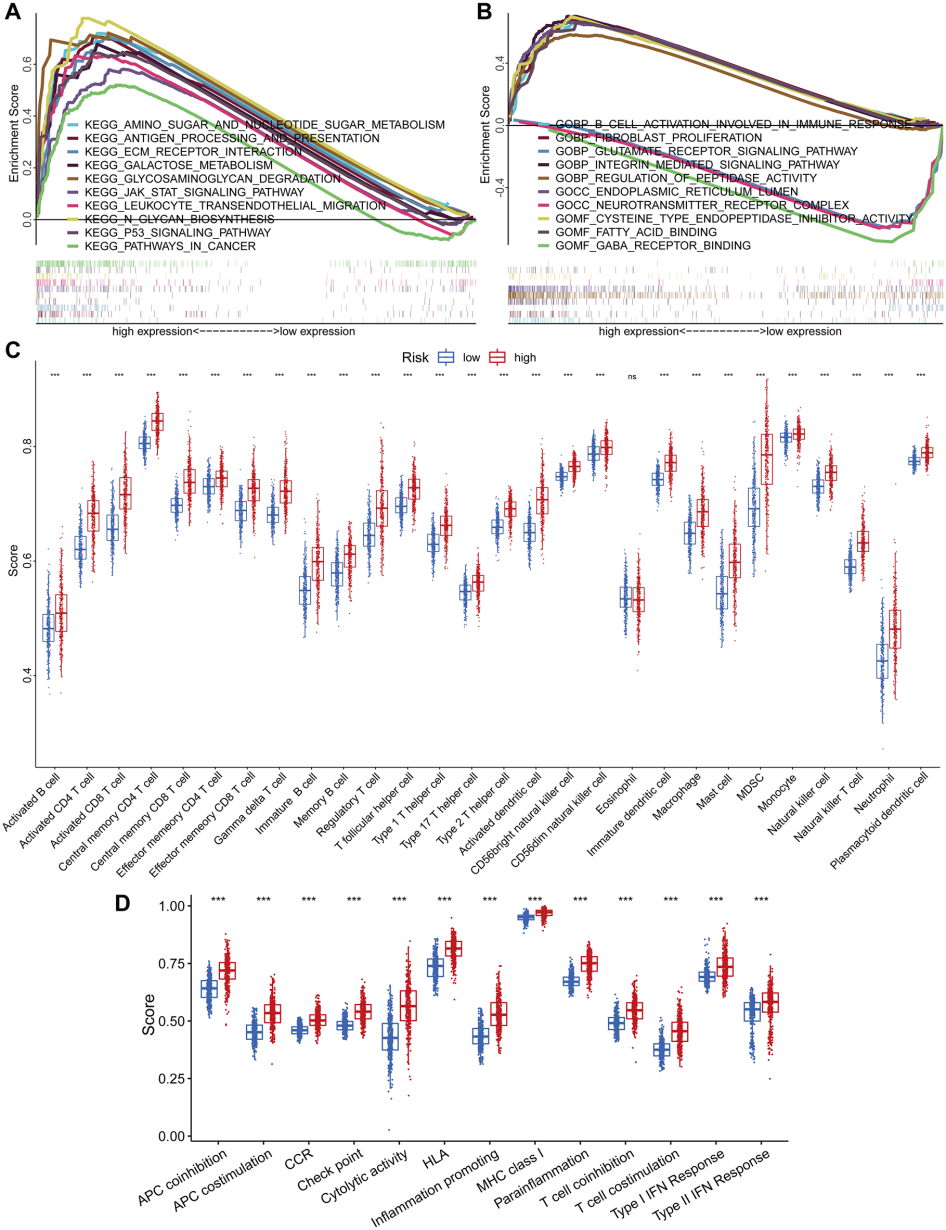
Risk model GSEA and immune correlation analysis. (**A**) GSEA-KEGG in the high-risk group. (**B**) GSEA-GO in the high-risk and low-risk groups. (**C**) Box plots of immune infiltration scores of 28 immune cells in the risk subgroup. (**D**) Box plots of immune function scores in risk subgroups. Red: high-risk group; blue: low-risk group. *p < 0.05, **p < 0.01, ***p < 0.001. KEGG: Kyoto Encyclopedia of Genes and Genomes; GOBP: biological process of gene ontology; GOCC: cell component of gene ontology; GOMF: molecular function of gene ontology.

In order to better understand the function of these seven genetic risk profiles, we divided 624 glioma patients into a high-risk group (n = 312) and a low-risk group (n = 312) based on their risk scores and performed GSEA on all genes in the gene expression profiles of the patients in the two groups, respectively (Supplementary Table 5). The results of the GSEA showed that several immune and tumor-related pathways were involved in the high-risk group, such as JAK-STAT signaling pathway, Antigen processing and presentation, Leukocyte transendothelial migration, B cell activation involved in immune response, integrin mediated signaling pathway and p53 signaling pathway. In addition, these genes are involved in pathways such as glycan biosynthesis and degradation and aminoglycan metabolism, such as N-Glycan Biosynthesis, glycosaminoglycan degradation, amino sugar and nucleotide sugar metabolism, Galactose metabolism and Aminoacyl-tRNA biosynthesis. The low-risk group was mainly enriched in neurotransmitter transmission-related pathways, such as glutamate receptor signaling pathway, GABA receptor binding and Neurotransmitter receptor complexes (Fig. 7A,B).

### Immuno-correlation analysis

**Figure 8:**
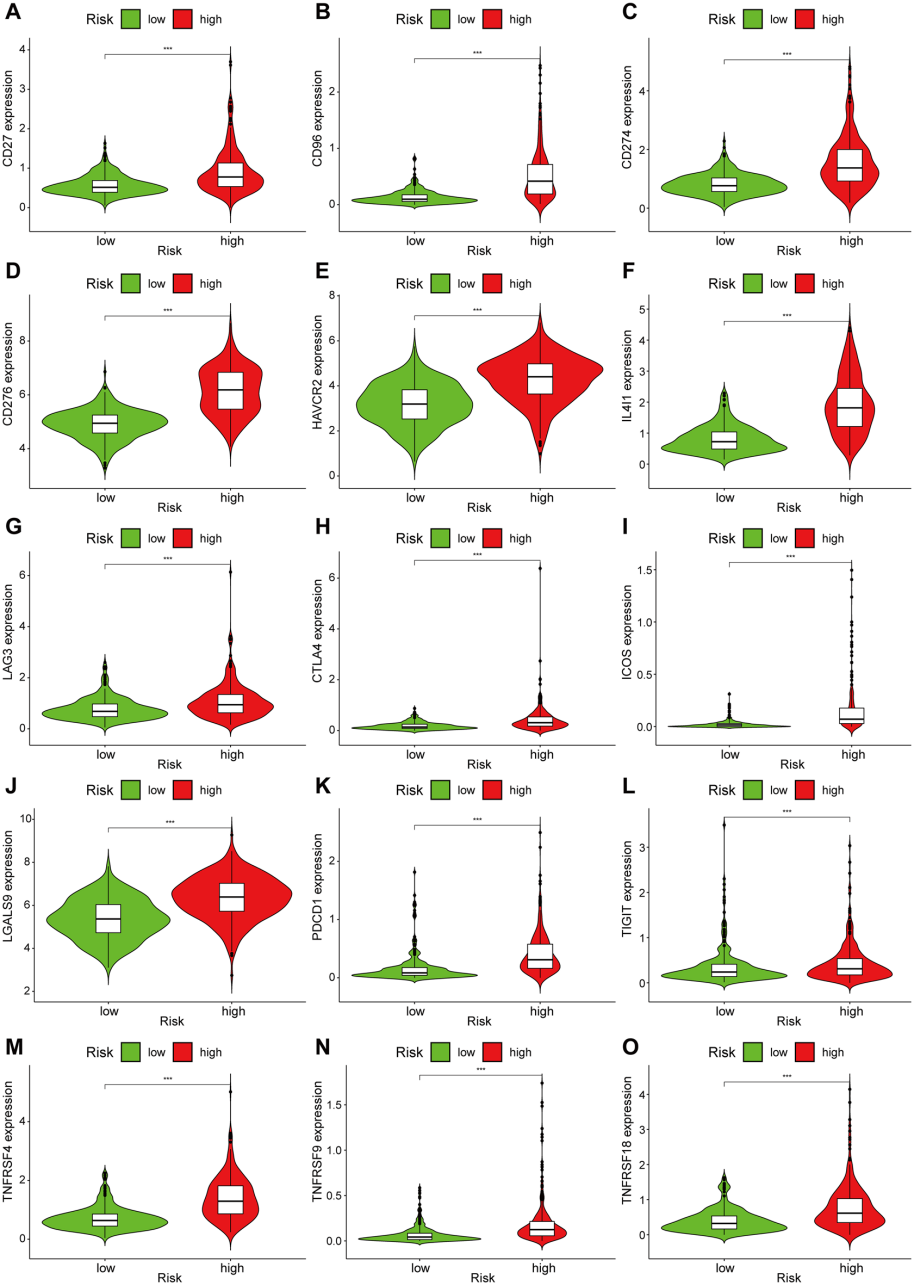
Differential analysis of immune checkpoint expression. (**A**–**O**) Violin plots of differential expression of *CD27*, *CD96*, *CD274*, *CD276*, *HAVCR2*, *IL4I1*, *LAG3*, *CTLA4*, *ICOS*, *LGALS9*, *PDCD1*, *TIGIT*, *TNFRSF4*, *TNFRSF9* and *TNFRSF18* immune checkpoints. *p < 0.05, **p < 0.01, ***p < 0.001. Red: high-risk group; green: low-risk group.

We performed immune cell and immune function analyses on the risk groups because the risk scores of the seven genes revealed in the enrichment analysis were linked to immune-related pathways that may play an important role in immune regulation. The findings revealed that all immune cells, except eosinophils, had higher immunological scores in the high-risk group of glioma patients than in the low-risk group, including macrophages, natural killer cells, and activated dendritic cells, and so on (Fig. 7C). The immune function evaluation also revealed that glioma patients in the high-risk group had significantly higher immunological scores than those in the low-risk group in a number of immune function modulations, such as Check point, Type I/II IFN Response, and Inflammation promoting (Fig. 7D). Thus, we compared the expression of immune checkpoints in two groups of glioma patients, which are currently undergoing clinical trials in several clinical trials, and the analysis showed that a variety of immune checkpoints were significantly more highly expressed in the high-risk group compared to the low-risk group (*p* < 0.05), including *CD27, CD96, CD274 (PD-L1), CD276 (B7-H3), CTLA4, HAVCR2 (TIM-3), ICOS, IL4I1, LAG3, LGALS9, PDCD1 (PD-1), TIGIT, TNFRSF4 (OX40), TNFRSF9* and *TNFRSF18* (*GITR*) (Fig. 8).

### The prognostic values of glycosylation related genes in glioma patients

**Figure 9:**
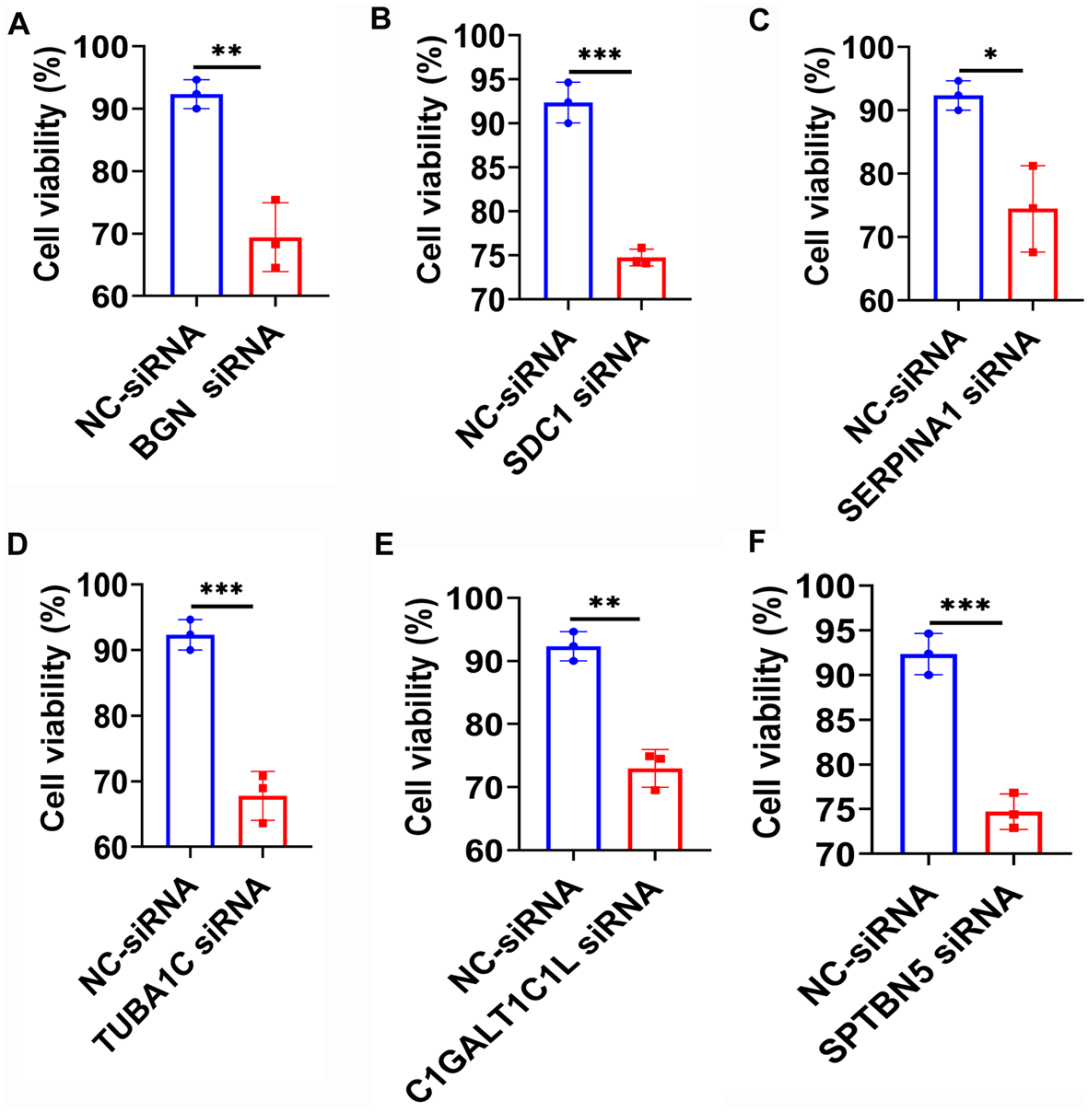
The role of glycosylation related genes in the viability of glioma U87 cells. (A-F) Cell viability of U87 tumour cells between control and *BGN*, *SDC1*, *SERPINA1*, *TUBA1C*, *C1GALT1C1L* and *SPTBN5*-silencing groups detected using CCK8 assay. N = 3, *p < 0.05, **p < 0.01, ***p < 0.001. NC: negative control.

**Figure 10:**
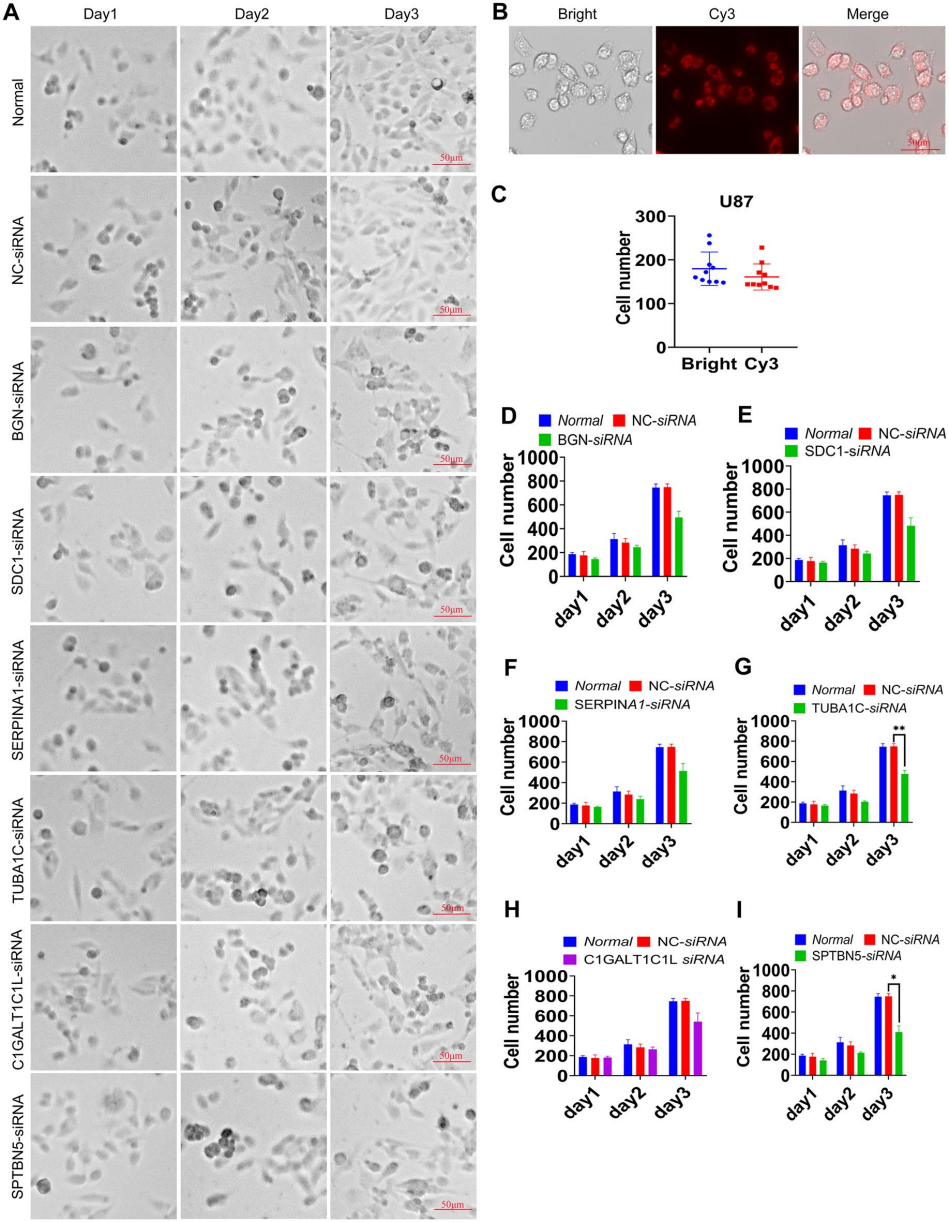
The role of glycosylation related genes in the viability of glioma U87 cells. (**A**) Bright field images showed that interfering with *BGN, SDC1, SERPINA1, TUBA1C, C1GALT1C1L* and *SPTBN5* factors inhibited U87 cell proliferation. (**B**,**C**) Negative control Cy3 transfection results showed siRNA could transfect almost all U87 cells. (**D**–**I**) The proliferation of U87 cells after interference with *BGN, SDC1, SERPINA1, TUBA1C, C1GALT1C1L,* and *SPTBN5* genes. Scale bar = 50 μm. N = 3, *p < 0.05, **p < 0.01. NC: negative control.

We analyzed the connections of these 7 genes to such clinicopathological factors as age, survival result, tumor grade, histological subtype, 1p/19q co-deletion status, radiotherapy status, MGMT promoter methylation, and IDH mutation status to see if there was a link between these genes and clinicopathological factors. *BGN*, *SDC1*, *SERPINA1*, *TUBA1C*, and a high risk score as risk variables were found to be positively connected with age (> 65), 1p/19q (non-codeletion), high-Grade, high-Histologic, IDH-status (Wildtype), MGMTp Methylated, Radiotherapy, and fustat (Dead). *GALNT13* as a protective factor correlated with age (< = 65), 1p/19q (codeletion), low-Grade, low-Histologic, IDH-status (Mutant), MGMTp Unmethylated, non-Radiotherapy and fustat (Alive) were positively correlated. *C1GALT1C1L* and *SPTBN5* were positively correlated as risk factors with high-Grade, IDH-status (Wildtype), MGMTp Methylated, Radiotherapy and fustat (Dead) (Supplementary Figs. 3–5). Hence, we examined the function of 6 unfavorable prognostic risk factors in the viability of glioma cells (U87 and U251 cells), which revealed that silencing *BGN, SDC1, SERPINA1, TUBA1C, C1GALT1C1L* and *SPTBN5* obviously inhibited the cell viability of U87 cells (Fig. 9A–F, p < 0.05, *p* < 0.01, *p* < 0.001) and U251 cells (Supplementary Fig. 6, *p* < 0.01, *p* < 0.001). In addition, bright field images showed that interfering with *BGN, SDC1, SERPINA1, TUBA1C, C1GALT1C1L* and *SPTBN5* factors inhibited the proliferation of U87 cells (Fig. 10) and U251 cells (Supplementary Fig. 7). At the third day, interference with *BGN*, *SDC1*, *SERPINA1*, *TUBA1C*, *C1GALT1C1L*, an*d SPTBN5* genes reduced the cell number of U87 cells (Fig. 10D–I), with statistical significance for *TUBA1C* and *SPTBN5* (*p* < 0.05, *p* < 0.01).

## Discussion

More alternatives for treating glioma are being developed all the time, and a combination of microsurgical treatment and adjuvant radiation therapy can kill or reduce local residual tumors as much as possible, although this can only enhance patient survival to a limited amount^15^. More precise subcategorization of treatment criteria has been introduced to the new edition of the WHO recommendations on glioma for patients with various prognoses and medication sensitivity, including isocitrate dehydrogenase IDH mutations, 1p/19q co-deletions, and MGMT promoter methylation^16^. IDH mutations and chromosome 1p/19q co-deletions are two of the most prevalent favorable genetic alterations in low-grade gliomas, and patients with IDH mutations and chromosome 1p/19q co-deletions have a better prognosis^14,17^. The addition of the adjuvant drug-alkylating agent temozolomide, which crosses the natural blood–brain barrier to reach intracranial lesions, leading to cellular DNA damage and eventually apoptosis, is the usual treatment for the most malignant glioblastomas; unfortunately, most patients do not benefit from drug therapy due to the presence of the MGMT promoter DNA repair gene^18^. Existing medicines only improve the prognosis of glioma patients to a limited extent, and additional effective biomarkers for individualized and accurate therapy are urgently needed.

Stable cell surface receptor signaling, cell–matrix interactions, antigen–antibody interactions, cellular infiltration, tumor invasion, and cell motility are all affected by glycosylation modifications^19^. A growing body of evidence suggests that abnormal protein glycosylation is a hallmark of cancer, and that tumor cell surface glycosylation characteristics are one of the key epigenetic changes that occur during the progression of malignant illness^20^. The major glycosylation alterations in gliomas include aberrant N-linked glycosylation and O-linked glycosylation on integrins and receptor tyrosine kinase, as well as aberrant glycoprotein sialylation. Overexpression of glycosyltransferases can promote glycan formation and increase glioma invasion and metastasis, and these glycosyltransferase target genes have great diagnostic and prognostic potential in gliomas^21,22^. We first used consistent clustering to divide glioma patients into two subgroups with optimal k = 2. PCA analysis revealed a clear separation between the two subgroups, so we used GO and KEGG enrichment analysis of differentially expressed genes between the two subgroups, where the KEGG pathway is involved in Proteoglycans in cancer, ECM–receptor interactionFocal adhesion, Cell adhesion molecules, and so on. Positive control of gliogenesis, collagen synthesis, glycans, and cell adhesion related biological processes such as "gliogenesis", "collagen fibril organization", "glycosaminoglycan binding" and "positive regulation of cell adhesion" are all engaged in GO enrichment analysis. These findings point to a significant biological function for differential glycosylation-related genes in the progression of glioma illness. In a previously constructed glioma prognostic model, apoptosis-related indicators showed good predictive value^23^. Currently, studies on the prognosis of gliomas have also shown that annexin A2 (*ANXA2*) is an unfavorable factor in the prognosis of gliomas, whereas annexin A2 is closely associated with glycosylation, so it is reasonable to believe that glycosylation and related genes play an important role in glioma development^24^. Another paper, using new machine learning algorithms, provides a good evaluation of the predictive efficacy of DNA methylation in the prognosis of glioma patients^25^. This is the first study to use risk profiles of glycosylation-related regulators to predict the prognosis of glioma patients, according to a review of the literature.

**Figure 11:**
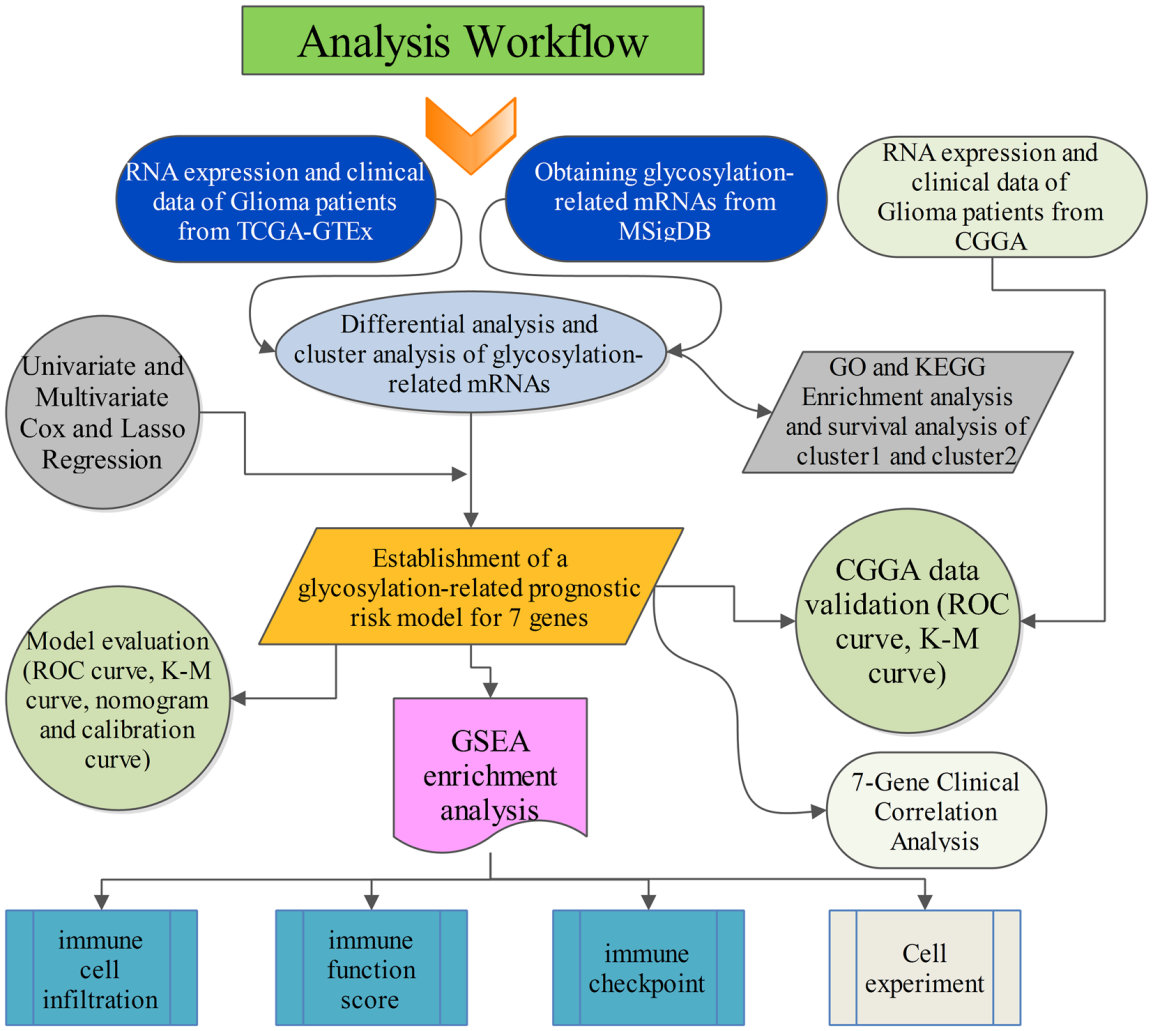
The flow chart demonstrates the main steps of this study.

Here, we examined the role of 552 genes related to glycosylation modifications in multiple genomes in glioma patients as a predictive factor. Using TCGA and GTEx database gene expression profiles and survival information, 44 glycosylation regulators associated with overall glioma survival were identified, and risk prognostic models based on seven genes (*BGN, C1GALT1C1L, GALNT13, SDC1, SERPINA1, SPTBN5, TUBA1C*) were developed and validated using CGGA database data for external data validation (Fig. 11). In both the prediction model cohort (TCGA-GTEx) and the external validation cohort (CGGA), Kaplan–Meier survival analysis revealed that patient survival was considerably worse in the high-risk group than in the low-risk group. The accuracy of the model was assessed using subject working characteristic curves, with AUCs of 0.892, 0.909, and 0.876 for the TCGA-GTEx cohort predicting 1-, 3-, and 5-year overall survival in glioma patients, respectively, and AUCs of 0.776, 0.829, and 0.826 for the CGGA validation cohort predicting 1-, 3-, and 5-year overall survival in glioma patients. After that, we ran univariate and multifactorial Cox regression analysis, finding that risk score was an independent risk factor for glioma prognosis. To help clinicians visually assess the survival of glioma patients, we used independent prognostic criteria to build a column line graph that could estimate a patient's death at 1, 3, and 5 years, and the calibration curve and C-index revealed that the column line graph was accurate. So far, we've determined that glycosylation-related genes in the model have a good predictive effect on glioma prognosis.

We discovered that these seven glycosylation genes are abnormally expressed in a variety of malignancies and are engaged in glycosylation modification-mediated carcinogenesis and immune modulation through a study of the literature. Biglycan (*BGN*) belongs to a family of tiny leucine-rich proteoglycoproteins that are extracellular matrix components and have a role in skeletal muscle growth and development, collagen fiber assembly, inflammatory modulation, and innate immunity. *BGN* was overexpressed in pancreatic cancer and inhibited cell development by blocking mitotic G1 phase of pancreatic cancer cells, while *BGN* was overexpressed in gastric cancer and positively linked with tumor cell repair, invasion, and migration^26,27^. Overexpression of *C1GALT1C1* in human colon cancer cells significantly enhances cell migration and invasion, with activation of the EMT signature as the underlying mechanism. *C1GALT1C1L* is a paralog of *C1GALT1C1*, a molecular chaperone located in the endoplasmic reticulum that plays a crucial role in proteinO-linked glycosylation^28^. *GALNT13* is a member of the N-acetylgalactosaminyltransferase family that is expressed particularly in neuronal cells and is responsible for the synthesis of O-glycan (Tn antigen)^29^. According to studies, *GALNT13* is highly expressed in lung cancer and is linked to a bad patient prognosis^30^. *SDC1* (syndecans), a member of the type I transmembrane protein family, has higher expression in gliomas than normal brain tissue and is related with tumor aggressiveness and poor prognosis. *SDC1* is also related to integrins and plays a role in cell adhesion and migration^31,32^. *SERPINA1* belongs to the serine protease inhibitor superfamily, and it has been studied as a target of abnormal protein fucosylation in pancreatic cancer. Elevated *SERPINA1* fucosylation was found to be positively correlated with TNM stage of pancreatic cancer, with high SERPINA1 indicating a poor prognosis^33^. *TUBA1C* is one of the α-microtubulin isoforms, which has a role in the development of many malignancies. *TUBA1C* expression was shown to be substantially expressed in low-grade gliomas and to be an independent risk factor for overall survival. *TUBA1C* expression was also found to be positively connected with the degree of infiltration of various immune cells in low-grade gliomas^34^. In addition, we conducted a GSEA enrichment analysis based on risk scores, with patients in the high-risk group being linked to pathways such as cytokine signaling, inflammatory responses, and immune regulation, as well as glycan synthesis and metabolic function, and those in the low-risk group being linked to synaptic receptors and neurotransmitter transmission. Finally, functional experiments were performed to evaluate the roles of risk genes in the cell viability of glioma cells, which demonstrated that silencing *BGN, SDC1, SERPINA1, TUBA1C, C1GALT1C1L* and *SPTBN5* could inhibit the growth and viability of glioma cells. These findings strengthened the prognostic potentials of our predictive signature in glioma.

Malignant cell proliferation and tumor migration are regulated by abnormal glycosylation changes, which also increase tumor-induced immunomodulatory responses^35^. Traditional combination therapy regimens have long been overwhelmed by central system malignancies due to natural anatomical barriers and tumor tissue heterogeneity, and thanks to high-throughput sequencing and the discovery of perivascular glial-lymphatic structures in the central system, immunotherapy and targeted therapy are once again the ultimate weapons clinicians have been waiting for^36^. Immunological checkpoint molecules, tumor-associated macrophages, and dendritic cell vaccines are currently the most appealing biomarkers, and clinical studies of targeted immune combination therapy for glioma are moving in that direction. Treatments targeting the immune checkpoints *IDO*, *CTLA-4*, and *PD-L1* reduced the number of tumor-infiltrating Tregs cells and improved mouse survival in a glioblast mouse model in previous studies, and combination targeted immunosuppressive therapy has high potential clinical value in high-grade malignant glioma^37^. Microglia, also known as CNS tumor-associated macrophages, are a key component of the glioma tumor microenvironment, and they can produce a range of growth factors and cytokines to control tumor proliferation and cancer cell migration in the glioma immune milieu^38^. Cytokines are important coordinators of the microenvironment in which gliomas develop, and thus cytokine regulators such as Suppressor of Cytokine Signaling 3 (*SOCS3*) play an active role in neural tissue development and cell proliferation. Thus, glycosylation may influence immune escape from gliomas by regulating the expression levels of certain cytokines in the body^39^. Immune responses against primary and metastatic cancers can be induced by dendritic cell tumor vaccines, and clinical trials have showed remarkable immunotherapeutic potential^6^. As a result, we compared the immune cell infiltration scores and immune function evaluations between the high-risk and low-risk groups, and the results revealed that a variety of immune cell infiltrations, such as Macrophage, activated dendritic cell, Regulatory T cell (Treg), and Natural killer cell, were increased in the high-risk group. In terms of cytolytic activity, checkpoint activation, and inflammation promotion, the immune function of the high-risk group outperformed that of the low-risk group. The expression of the more well-known immune-related targets in glioma, such as *CTLA4*, *PDCD1* (*PD-1*), *CD274* (*PD-L1*), *LAG-3*, and *CD47*, was then examined. Anti-*CTLA-4*, anti-*PD-1*, and anti-*PD-L1* therapy were found to improve the antitumor immune response of glioblastoma patients treated with targeted *CD73*. It is suggested that tyrosine metabolizing enzymes regulate adaptive immune processes in gliomas, which are mechanistically related to the remodeling of multiple metabolizing enzymes that induce the expression of programmed death ligand 1^40^. *LAG3* is found on activated immune cells and, like *PD-1*, stimulates tumor cell immune escape. *CD47*, also known as "integrin-related protein," is thought to enhance the invasion and progression of high-grade glioma. Anti-*LAG-3* and anti-*CD47* inhibitors could be used as immunotherapy modulators^41,42^. Researchers have proposed multi-immune checkpoint combination therapy and immunotherapy in combination with other targeted pathways in response to the tendency of single-target therapy to relapse and develop drug resistance^43^. These findings led us to believe that risk profiles based on glycosylation modification modifiers linked to glioma tumor formation and immune modulation have a fair chance of accurately predicting glioma patient prognosis.

## Conclusions

Collectively, we discovered that gene sets associated to glycosylation metabolism can discriminate clinical, pathological, and molecular aspects of gliomas. The established glycosylation-related risk profiles were independent prognostic predictors of glioma. These findings were well externally validated in the CGGA database. Combined with biological experiments at cellular level, this study provides a new glycosylation-related risk profile for predicting glioma prognosis, aids in the investigation of the occurrence and progression of glioma, and identifies novel targets for glioma diagnosis and treatment.

### Supplementary Information

Supplementary Information 1.

Supplementary Table 1.

Supplementary Table 2.

Supplementary Table 3.

Supplementary Table 4.

Supplementary Table 5.

## Data Availability

The original data used to support the findings of this study are included within the supplementary information files.

## References

[1] Weller M, Wick W, Aldape K (2015). Glioma. Nat. Rev. Dis. Primers.

[2] Pi Y, Fang CL, Su ZY (2022). Protein phosphorylation: A potential target in glioma development. Ibrain.

[3] Velásquez C, Mansouri S, Mora C (2019). Molecular and clinical insights into the invasive capacity of glioblastoma cells. J. Oncol..

[4] Ostrom QT, Cioffi G, Waite K, Kruchko C, Barnholtz-Sloan JS (2021). CBTRUS statistical report: Primary brain and other central nervous system tumors diagnosed in the United States in 2014–2018. Neuro Oncol..

[5] Chai Y, Liu S, Xie MX (2021). Interaction among long non-coding RNA, micro-RNA and mRNA in glioma. Ibrain.

[6] Chen R, Smith-Cohn M, Cohen AL, Colman H (2017). Glioma subclassifications and their clinical significance. Neurotherapeutics.

[7] Eichler J (2019). Protein glycosylation. Curr. Biol..

[8] Häuselmann I, Borsig L (2014). Altered tumor-cell glycosylation promotes metastasis. Front. Oncol..

[9] Schjoldager KT, Narimatsu Y, Joshi HJ, Clausen H (2020). Global view of human protein glycosylation pathways and functions. Nat. Rev. Mol. Cell Biol..

[10] Fuster MM, Esko JD (2005). The sweet and sour of cancer: Glycans as novel therapeutic targets. Nat. Rev. Cancer.

[11] Cheray M, Petit D, Forestier L (2011). Glycosylation-related gene expression is linked to differentiation status in glioblastomas undifferentiated cells. Cancer Lett..

[12] Baro M, Lopez Sambrooks C, Quijano A, Saltzman WM, Contessa J (2019). Oligosaccharyltransferase inhibition reduces receptor tyrosine kinase activation and enhances glioma radiosensitivity. Clin. Cancer Res..

[13] Kanehisa M, Furumichi M, Sato Y, Kawashima M, Ishiguro-Watanabe M (2023). KEGG for taxonomy-based analysis of pathways and genomes. Nucleic Acids Res..

[14] Brandner S, McAleenan A, Jones HE (2021). Diagnostic accuracy of 1p/19q codeletion tests in oligodendroglioma: A comprehensive meta-analysis based on a Cochrane systematic review. Neuropathol. Appl. Neurobiol..

[15] Shah JL, Li G, Shaffer JL (2018). Stereotactic radiosurgery and hypofractionated radiotherapy for glioblastoma. Neurosurgery.

[16] Louis DN, Perry A, Wesseling P (2021). The 2021 WHO classification of tumors of the central nervous system: A summary. Neuro Oncol..

[17] Turkalp Z, Karamchandani J, Das S (2014). IDH mutation in glioma: New insights and promises for the future. JAMA Neurol..

[18] Hegi ME, Diserens AC, Gorlia T (2005). MGMT gene silencing and benefit from temozolomide in glioblastoma. N. Engl. J. Med..

[19] Pinho SS, Reis CA (2015). Glycosylation in cancer: Mechanisms and clinical implications. Nat. Rev. Cancer.

[20] Stowell SR, Ju T, Cummings RD (2015). Protein glycosylation in cancer. Annu. Rev. Pathol..

[21] Veillon L, Fakih C, Abou-El-Hassan H, Kobeissy F, Mechref Y (2018). Glycosylation changes in brain cancer. ACS Chem. Neurosci..

[22] Cuello HA, Ferreira GM, Gulino CA, Toledo AG, Segatori VI, Gabri MR (2020). Terminally sialylated and fucosylated complex N-glycans are involved in the malignant behavior of high-grade glioma. Oncotarget.

[23] Zhang M, Cheng Y, Xue Z, Sun Q, Zhang J (2021). A novel pyroptosis-related gene signature predicts the prognosis of glioma through immune infiltration. BMC Cancer.

[24] Ma K, Chen X, Liu W (2021). ANXA2 is correlated with the molecular features and clinical prognosis of glioma, and acts as a potential marker of immunosuppression. Sci. Rep..

[25] Tian J, Zhu M, Ren Z (2022). Deep learning algorithm reveals two prognostic subtypes in patients with gliomas. BMC Bioinform..

[26] Weber CK, Sommer G, Michl P (2001). Biglycan is overexpressed in pancreatic cancer and induces G1-arrest in pancreatic cancer cell lines. Gastroenterology.

[27] Hu L, Duan YT, Li JF (2014). Biglycan enhances gastric cancer invasion by activating FAK signaling pathway. Oncotarget.

[28] Gao T, Du T, Hu X (2020). Cosmc overexpression enhances malignancies in human colon cancer. J. Cell. Mol. Med..

[29] Zhang Y, Iwasaki H, Wang H (2003). Cloning and characterization of a new human UDP-N-acetyl-alpha-D-galactosamine:polypeptide N-acetylgalactosaminyltransferase, designated pp-GalNAc-T13, that is specifically expressed in neurons and synthesizes GalNAc alpha-serine/threonine antigen. J. Biol. Chem..

[30] Nogimori K, Hori T, Kawaguchi K (2016). Increased expression levels of ppGalNAc-T13 in lung cancers: Significance in the prognostic diagnosis. Int. J. Oncol..

[31] Watanabe A, Mabuchi T, Satoh E (2006). Expression of syndecans, a heparan sulfate proteoglycan, in malignant gliomas: participation of nuclear factor-kappaB in upregulation of syndecan-1 expression. J. Neurooncol..

[32] Marsico G, Russo L, Quondamatteo F, Pandit A (2018). Glycosylation and integrin regulation in cancer. Trends Cancer..

[33] Wu CC, Lu YT, Yeh TS, Chan YH, Dash S, Yu JS (2021). Identification of fucosylated SERPINA1 as a novel plasma marker for pancreatic cancer using lectin affinity capture coupled with iTRAQ-Based quantitative glycoproteomics. Int. J. Mol. Sci..

[34] Zhu H, Hu X, Gu L (2021). TUBA1C is a prognostic marker in low-grade glioma and correlates with immune cell infiltration in the tumor microenvironment. Front. Genet..

[35] Läubli H, Borsig L (2019). Altered cell adhesion and glycosylation promote cancer immune suppression and metastasis. Front Immunol..

[36] Rasmussen MK, Mestre H, Nedergaard M (2018). The glymphatic pathway in neurological disorders. Lancet Neurol..

[37] Wainwright DA, Chang AL, Dey M (2014). Durable therapeutic efficacy utilizing combinatorial blockade against IDO, CTLA-4, and PD-L1 in mice with brain tumors. Clin. Cancer Res..

[38] Hambardzumyan D, Gutmann DH, Kettenmann H (2016). The role of microglia and macrophages in glioma maintenance and progression. Nat. Neurosci..

[39] Crall JJ (1986). Preferences for treatment by dental specialists. J. Dent. Educ..

[40] Wang JY, Dai XT, Gao QL (2023). Tyrosine metabolic reprogramming coordinated with the tricarboxylic acid cycle to drive glioma immune evasion by regulating PD-L1 expression. Ibrain.

[41] Daubon T, Léon C, Clarke K (2019). Deciphering the complex role of thrombospondin-1 in glioblastoma development. Nat. Commun..

[42] Maruhashi T, Sugiura D, Okazaki IM, Okazaki T (2020). LAG-3: From molecular functions to clinical applications. J. Immunother. Cancer.

[43] Yang K, Wu Z, Zhang H (2022). Glioma targeted therapy: Insight into future of molecular approaches. Mol. Cancer.

