# Supplementary material for: Predictive potentials of glycosylation-related genes in glioma prognosis and their correlation with immune infiltration
Source: Sci Rep. 2024 Feb 23;14:4478. doi: 10.1038/s41598-024-51973-0 (PMC10891078; doi:10.1038/s41598-024-51973-0)
Supplement: Supplementary file 1 — Supplementary Information 1. [file 41598_2024_51973_MOESM1_ESM.docx]

**Supplementary figure and table legends**

**Supplementary Figure 1:** 44 genes associated with OS of glioma patients (*P* < 0.05) with HR > 1 (red), and HR < 1 (green).

**Supplementary Figure 2:** Heat map of expression of seven glycosylation-related genes combined with clinicopathological factors in high-risk and low-risk groups. * *P* < 0.05, ** *P* < 0.01 and *** *P* < 0.001.

**Supplementary Figure 3:** A-C: Correlation of BGN, GALNT13 and SDC1 with 1p/19q, Age, fustat, Grade, Histologic, IDH-status, MGMTp Methylated and Radiotherapy.

**Supplementary Figure 4:** A-C: Correlation of SERPINA1, TUBA1C and Risk score with 1p/19q, Age, fustat, Grade, Histologic, IDH-status, MGMTp Methylated and Radiotherapy.

**Supplementary Figure 5:** A-B: Correlation of C1GALT1C1L and SPTBN5 with 1p/19q, Age, fustat, Grade, Histologic, IDH-status, MGMTp Methylated and Radiotherapy.


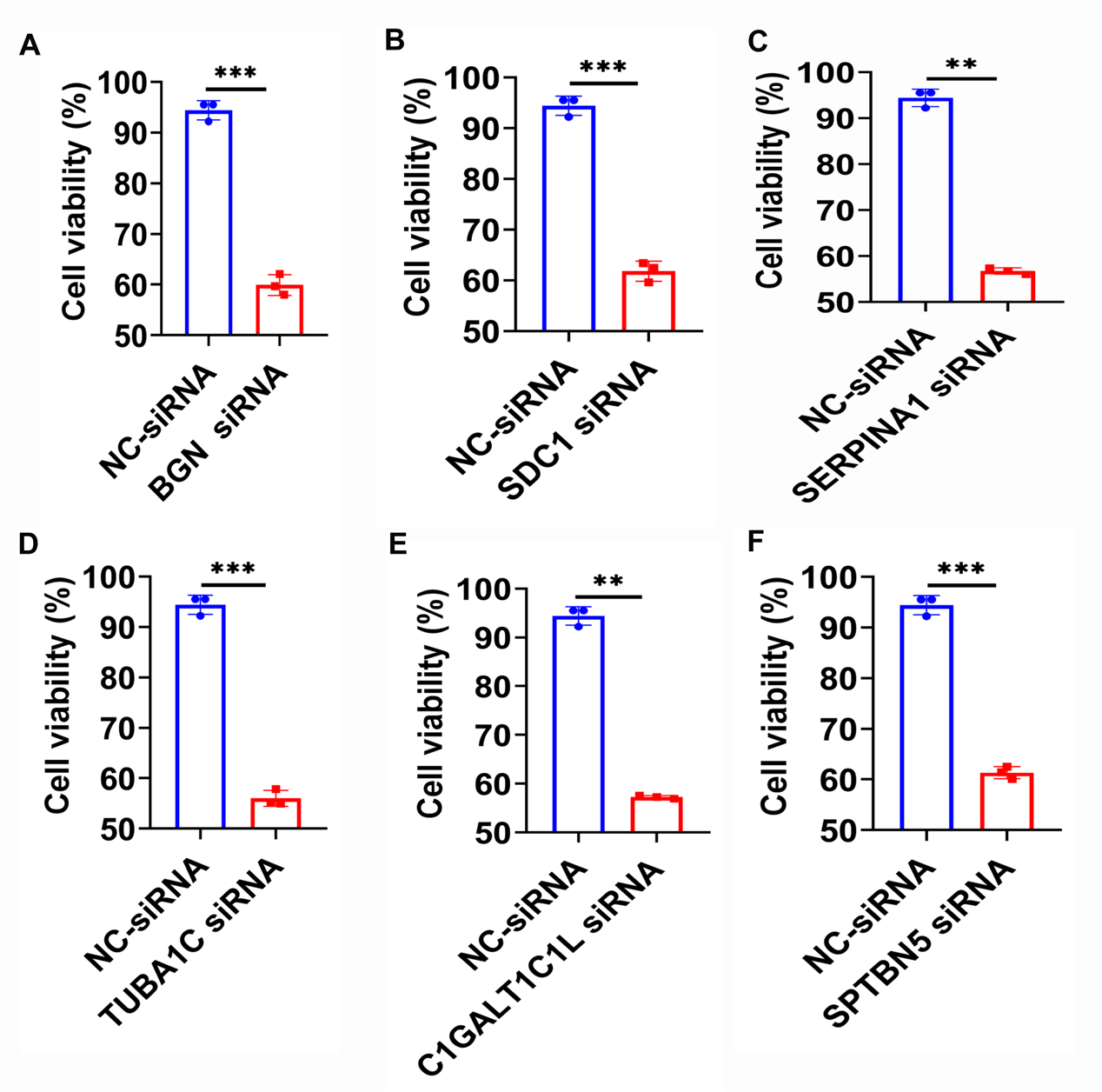


**Supplementary Figure 6:** **The role of glycosylation related genes in the viability of glioma U251 cells.** (A-F) Cell viability of U251 tumour cells between control and *BGN*, *SDC1*, *SERPINA1*, *TUBA1C*, *C1GALT1C1L* and *SPTBN5*-silencing groups detected using CCK8 assay. N = 3, ***p* < 0.01, ****p* < 0.001.


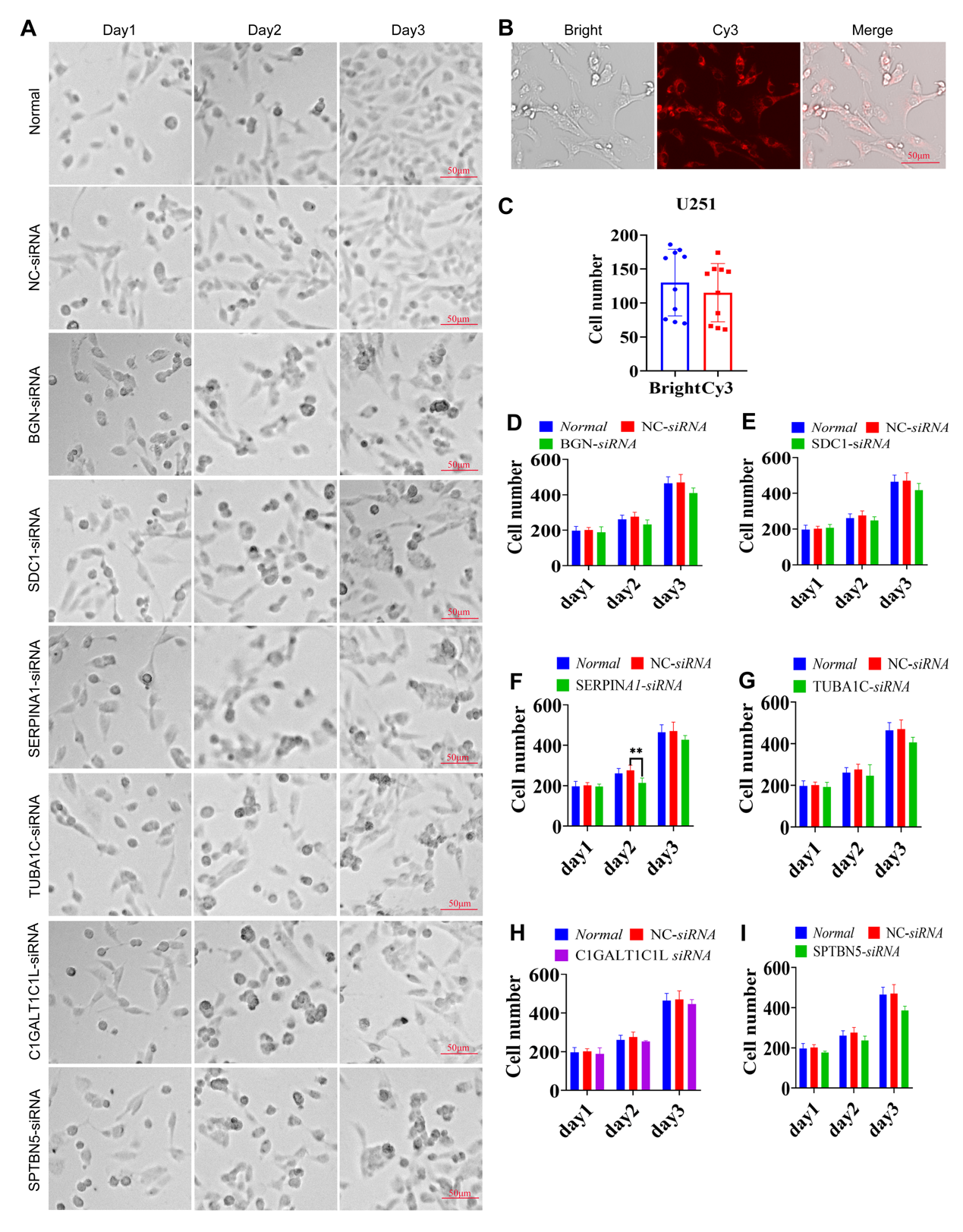


**Supplementary Figure 7 The role of glycosylation related genes in the viability of glioma U251 cells.** (A) Bright field images showed that interfering with *BGN, SDC1, SERPINA1, TUBA1C, C1GALT1C1L* and *SPTBN5* factors inhibited U251 cell proliferation. (B, C) Negative control Cy3 transfection results showed siRNA could transfect almost all U251 cells. (D-I) The proliferation of U251 cells after interference with *BGN, SDC1, SERPINA1, TUBA1C, C1GALT1C1L,* and *SPTBN5* genes. Scale bar = 50 μm. N = 3, ***p* < 0.01.

**Supplementary table 1:** The list of genes involved in GO functions.

**Supplementary table 2:** Expression levels of differentially expressed genes.

**Supplementary table 3:** GO terms involved by differentially expressed genes.

**Supplementary table 4:** Three Machine Learning Methods for Screening Differentially Expressed Genes for Constructing Prognostic Models.

**Supplementary table 5:** GSEA enrichment based on sample risk grouping.
