# Supplementary material for: Predictive potentials of glycosylation-related genes in glioma prognosis and their correlation with immune infiltration
Source: Sci Rep. 2024 Feb 23;14:4478. doi: 10.1038/s41598-024-51973-0 (PMC10891078; doi:10.1038/s41598-024-51973-0)
Supplement: Supplementary file 4 — Supplementary Table 3. [file 41598_2024_51973_MOESM4_ESM.docx]

Supplementary Table S3 GO and KEGG

| Ontology | ID | Description | GeneRatio | BgRatio | pvalue | p.adjust | qvalue |
| --- | --- | --- | --- | --- | --- | --- | --- |
| BP | GO:0030199 | collagen fibril organization | 14/491 | 54/18670 | 7.80e-11 | 1.11e-07 | 8.77e-08 |
| BP | GO:0045785 | positive regulation of cell adhesion | 31/491 | 403/18670 | 1.04e-07 | 4.93e-05 | 3.91e-05 |
| BP | GO:0050808 | synapse organization | 31/491 | 408/18670 | 1.37e-07 | 5.85e-05 | 4.63e-05 |
| BP | GO:0048708 | astrocyte differentiation | 12/491 | 81/18670 | 1.30e-06 | 2.42e-04 | 1.91e-04 |
| BP | GO:0042063 | gliogenesis | 23/491 | 290/18670 | 2.80e-06 | 4.27e-04 | 3.38e-04 |
| CC | GO:0062023 | collagen-containing extracellular matrix | 74/510 | 406/19717 | 2.48e-41 | 1.11e-38 | 8.12e-39 |
| CC | GO:0005604 | basement membrane | 19/510 | 95/19717 | 3.64e-12 | 5.43e-10 | 3.97e-10 |
| CC | GO:0005581 | collagen trimer | 17/510 | 87/19717 | 7.42e-11 | 8.29e-09 | 6.07e-09 |
| CC | GO:0005788 | endoplasmic reticulum lumen | 31/510 | 309/19717 | 1.29e-10 | 1.15e-08 | 8.42e-09 |
| CC | GO:0098644 | complex of collagen trimers | 9/510 | 19/19717 | 3.55e-10 | 2.64e-08 | 1.93e-08 |
| MF | GO:0005201 | extracellular matrix structural constituent | 34/475 | 163/17697 | 6.67e-21 | 4.32e-18 | 3.56e-18 |
| MF | GO:0005539 | glycosaminoglycan binding | 29/475 | 229/17697 | 3.98e-12 | 1.29e-09 | 1.06e-09 |
| MF | GO:0008201 | heparin binding | 22/475 | 169/17697 | 9.60e-10 | 2.07e-07 | 1.71e-07 |
| MF | GO:0005518 | collagen binding | 9/475 | 67/17697 | 7.20e-05 | 0.002 | 0.001 |
| MF | GO:0032395 | MHC class II receptor activity | 4/475 | 10/17697 | 9.46e-05 | 0.002 | 0.002 |
| KEGG | hsa05150 | Staphylococcus aureus infection | 16/229 | 96/8076 | 9.14e-09 | 2.33e-06 | 1.98e-06 |
| KEGG | hsa04512 | ECM-receptor interaction | 15/229 | 88/8076 | 1.97e-08 | 2.51e-06 | 2.13e-06 |
| KEGG | hsa04510 | Focal adhesion | 19/229 | 201/8076 | 3.81e-06 | 1.41e-04 | 1.20e-04 |
| KEGG | hsa04514 | Cell adhesion molecules | 16/229 | 149/8076 | 4.44e-06 | 1.41e-04 | 1.20e-04 |
| KEGG | hsa05205 | Proteoglycans in cancer | 19/229 | 205/8076 | 5.09e-06 | 1.44e-04 | 1.23e-04 |
