# Supplementary material for: Predictive potentials of glycosylation-related genes in glioma prognosis and their correlation with immune infiltration
Source: Sci Rep. 2024 Feb 23;14:4478. doi: 10.1038/s41598-024-51973-0 (PMC10891078; doi:10.1038/s41598-024-51973-0)
Supplement: Supplementary file 5 — Supplementary Table 4. [file 41598_2024_51973_MOESM5_ESM.docx]

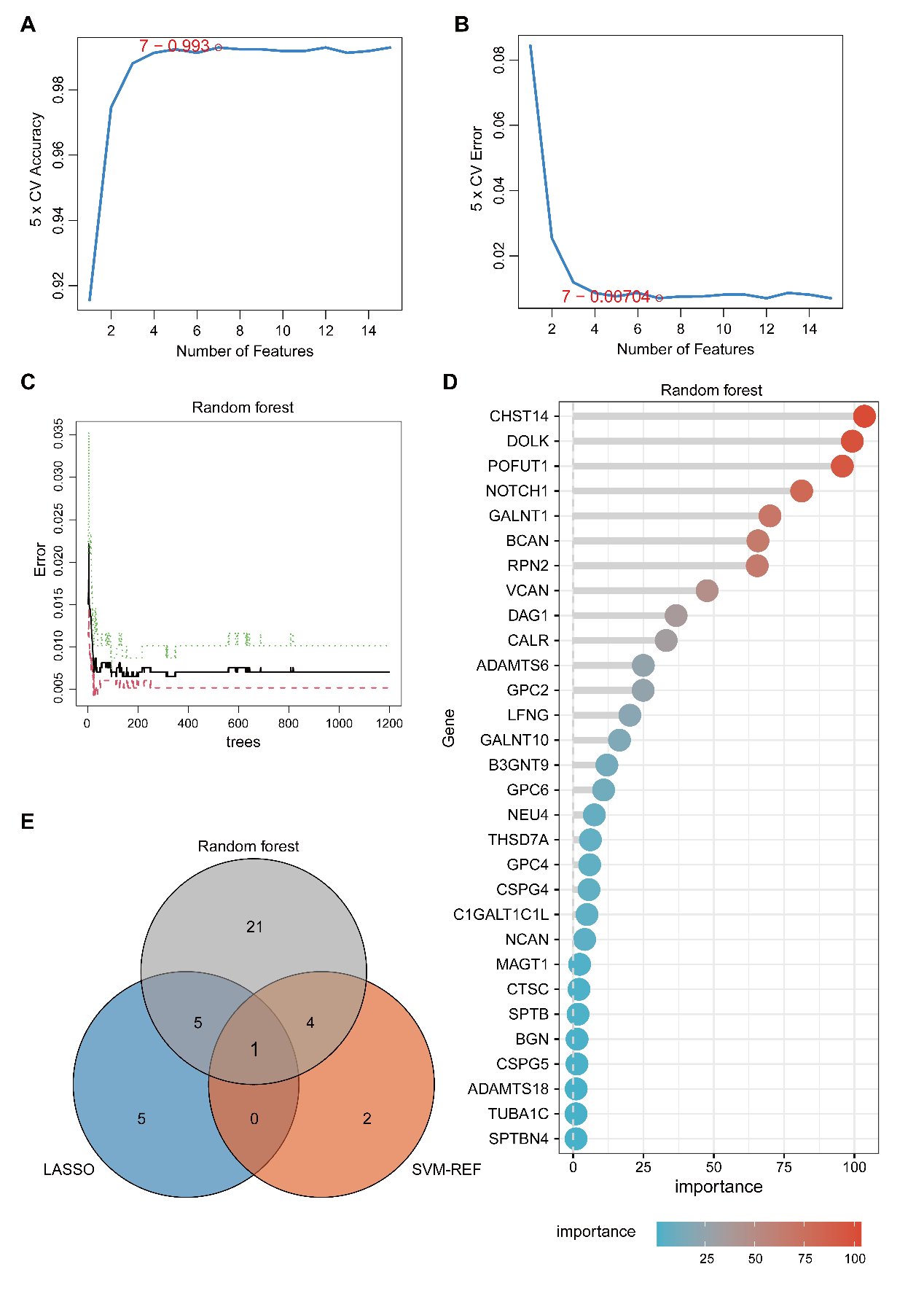


**Supplementary table 4:** Three Machine Learning Methods for Screening Differentially Expressed Genes for Constructing Prognostic Models.

A-B: SVM-RFE algorithm screened diagnostic markers. The red circle indicates the number of factors corresponding to the lowest error rate. C: RF algorithm screened the diagnostic markers. The x-axis represents the number of decision trees, and the y-axis represents the error rate. D: The top 30 genes sorted based on the RF classifier Gini efficient method. The x-axis represents importance, and the y-axis represents genes. E: The overlapping genes screened out by Lasso, RF and SVM-RFE algorithms.

| **Gene** | **Gene** | **importance** | **FeatureName** | **FeatureID** | **AvgRank** |
| --- | --- | --- | --- | --- | --- |
| BGN | CHST14 | 103.5722 | DAG1 | 12 | 5.3 |
| C1GALT1C1L | DOLK | 99.24334 | NOTCH1 | 30 | 5.4 |
| CTSC | POFUT1 | 95.68985 | CHST14 | 8 | 6.1 |
| FMOD | NOTCH1 | 81.21582 | BCAN | 4 | 7 |
| GALNT13 | GALNT1 | 69.97732 | GALNT12 | 18 | 7.3 |
| NEU4 | BCAN | 65.68544 | NEU4 | 29 | 8.7 |
| RPN2 | RPN2 | 65.43045 | SPON1 | 35 | 11.9 |
| SDC1 | VCAN | 47.61779 | ADAMTS18 | 1 | 12.5 |
| SERPINA1 | DAG1 | 36.59994 | POFUT1 | 31 | 15.5 |
| SPTBN5 | CALR | 33.05907 | DOLK | 13 | 15.7 |
| TUBA1C | ADAMTS6 | 24.94159 | SERPINA1 | 34 | 15.7 |
|  | GPC2 | 24.8862 | SPTBN5 | 38 | 16 |
|  | LFNG | 20.15217 | RPN2 | 32 | 17.3 |
|  | GALNT10 | 16.46108 | HSPG2 | 24 | 18.3 |
|  | B3GNT9 | 11.9901 | GPC2 | 20 | 18.7 |
|  | GPC6 | 10.82037 | GALNT1 | 16 | 19.6 |
|  | NEU4 | 7.539832 | F5 | 14 | 20.8 |
|  | THSD7A | 6.117268 | LUM | 26 | 20.8 |
|  | GPC4 | 5.860766 | THBS2 | 41 | 20.8 |
|  | CSPG4 | 5.613643 | THSD7A | 42 | 22 |
|  | C1GALT1C1L | 4.91112 | C1GALT1C1L | 6 | 23.1 |
|  | NCAN | 4.073278 | FMOD | 15 | 23.5 |
|  | MAGT1 | 2.28156 | GALNT10 | 17 | 23.5 |
|  | CTSC | 2.081251 | BGN | 5 | 23.9 |
|  | SPTB | 1.730517 | SDC1 | 33 | 24.9 |
|  | BGN | 1.382846 | B3GNT9 | 3 | 26 |
|  | CSPG5 | 1.341944 | NCAN | 28 | 26.1 |
|  | ADAMTS18 | 1.07491 | CSPG5 | 10 | 26.5 |
|  | TUBA1C | 1.0175 | GPC4 | 22 | 26.9 |
|  | SPTBN4 | 1.016368 | CALR | 7 | 27.7 |
|  | F5 | 1.011115 | GPC3 | 21 | 27.7 |
|  | GALNT12 | 0.857981 | GPC6 | 23 | 28.3 |
|  | SDC1 | 0.816157 | SPTBN4 | 37 | 28.3 |
|  | SPTBN5 | 0.801485 | ADAMTS6 | 2 | 28.4 |
|  | ST8SIA3 | 0.766745 | LFNG | 25 | 28.9 |
|  | SERPINA1 | 0.726054 | CSPG4 | 9 | 30.9 |
|  | LUM | 0.570463 | VCAN | 44 | 31.3 |
|  | GPC3 | 0.513124 | MAGT1 | 27 | 33.1 |
|  | SPON1 | 0.409532 | SPTB | 36 | 33.2 |
|  | THBS2 | 0.347669 | GALNT13 | 19 | 33.6 |
|  | GALNT13 | 0.326448 | TUBA1C | 43 | 34.8 |
|  | FMOD | 0.310891 | CTSC | 11 | 36.4 |
|  | THBS1 | 0.307983 | THBS1 | 40 | 37.5 |
|  | HSPG2 | 0.253621 | ST8SIA3 | 39 | 40.1 |

**Venn gene name**

| **Only LASSO** | **Only RandomForest** | | **Only SVM-REF** | **LASSO AND RandomForest NOT SVM-REF** | **LASSO AND SVM-REF NOT RandomForest** | **RandomForest AND SVM-REF NOT LASSO** | **LASSO AND RandomForest AND SVM-REF** | |
| --- | --- | --- | --- | --- | --- | --- | --- | --- |
| FMOD | | GALNT1 | GALNT12 | BGN | SERPINA1 | CHST14 | NEU4 |  |
| GALNT13 | | VCAN | SPON1 | C1GALT1C1L | SPTBN5 | DOLK |  |  |
| SDC1 | | CALR |  | CTSC |  | POFUT1 |  |  |
|  | | ADAMTS6 |  | RPN2 |  | NOTCH1 |  |  |
|  | | GPC2 |  | TUBA1C |  | BCAN |  |  |
|  | | LFNG |  |  |  | DAG1 |  |  |
|  | | GALNT10 |  |  |  | ADAMTS18 | |  |
|  | | B3GNT9 |  |  |  |  |  |  |
|  | | GPC6 |  |  |  |  |  |  |
|  | | THSD7A |  |  |  |  |  |  |
|  | | GPC4 |  |  |  |  |  |  |
|  | | CSPG4 |  |  |  |  |  |  |
|  | | NCAN |  |  |  |  |  |  |
|  | | MAGT1 |  |  |  |  |  |  |
|  | | SPTB |  |  |  |  |  |  |
|  | | CSPG5 |  |  |  |  |  |  |
|  | | SPTBN4 |  |  |  |  |  |  |
|  | | F5 |  |  |  |  |  |  |
